# The Evolution of Morphospace in Phytophagous Scarab Chafers: No Competition - No Divergence?

**DOI:** 10.1371/journal.pone.0098536

**Published:** 2014-05-29

**Authors:** Jonas Eberle, Renier Myburgh, Dirk Ahrens

**Affiliations:** 1 Zoologisches Forschungsmuseum Alexander Koenig, Centre of Taxonomy and Evolutionary Research, Bonn, Germany; 2 Department of Entomology, Natural History Museum, London, United Kingdom; 3 Division of Infectious Diseases and Hospital Epidemiology, University Hospital of Zürich, Zurich, Switzerland; CNRS, Université de Bourgogne, France

## Abstract

Body shape reflects species' evolution and mediates its role in the environment as it integrates gene expression, life style, and structural morphology. Its comparative analysis may reveal insight on what shapes shape, being a useful approach when other evidence is lacking. Here we investigated evolutionary patterns of body shape in the highly diverse phytophagous chafers (Scarabaeidae: Pleurosticti), a polyphagous group utilizing different parts of angiosperms. Because the reasons of their successful diversification are largely unknown, we used a phylogenetic tree and multivariate analysis on twenty linear measurements of body morphology including all major Pleurosticti lineages to infer patterns of morphospace covariation and divergence. The chafer's different feeding types resulted to be not distinguishable in the described morphospace which was largely attributed to large occupancy of the morphospace of some feeding types and to multiple convergences of feeding behavior (particularly of anthophagy). Low correlation between molecular and morphological rates of evolution, including significant rate shifts for some lineages, indicated directed selection within feeding types. This is supported by morphospace divergence within feeding types and convergent evolution in Australian Melolonthinae. Traits driving morphospace divergence were extremities and traits linked with locomotion behavior, but also body size. Being highly adaptive for burrowing and locomotion these traits showed major changes in the evolution of pleurostict scarabs. These activities also affected another trait, the metacoxal length, which is highly influenced by key innovations of the metacoxa (extended mesal process, secondary closure) particularly in one lineage, the Sericini. Significant shape divergence between major lineages and a lack of strong differentiation among closely related lineages indicated that the question about the presence or absence of competition-derived directed selection needs to be addressed for different time scales. Striking divergence between some sister lineages at their origin revealed strong driven selection towards morphospace divergence, possibly linked with resource partitioning.

## Introduction

Phytophagous scarabs (Coleoptera: Scarabaeidae) are a very diverse group of some 25,000 described species of beetles [1] which includes more than two thirds of all species in the superfamily Scarabaeoidea. Their monophyly is supported by a number of distinct morphological synapomorphies [2]–[4]. Early in taxonomic history, they were recognized as a group called Pleurosticti [5]. Pleurosticts are usually subdivided into four major subfamilies including: Dynastinae, Rutelinae, Melolonthinae, and Cetoniinae, plus several other small groups [6]. Most species are highly polyphagous, with the adults generally feeding on leaves, flowers or pollen of a wide range of plant taxa, and the larvae primarily feeding on soil humus, living roots, or decaying wood. Because of this polyphagy, their tremendous diversity cannot be explained by insect-host plant co-diversification, a widely accepted hypothesis for the great species diversity in phytophagous insects [7]–[9], hence alternative hypotheses are needed that may explain their successful diversification.

For many groups of organisms it was argued that niche partitioning, as a result of competition, leads to a positive relationship between species richness and the ecomorphological diversity of animal assemblages [10]. It is well known and widely accepted that resource partitioning is one of the most important factors in scarab biology leading to profound structural changes and adaptations for particular feeding functions or foraging behavior [3].

Scholtz and Chown [11] proposed a substantial shift in Scarabaeoidea biology with the use of living plant material as a food resource instead of dead or decayed organic matter. They assumed that the massive radiation of the main pleurostict lineages (Melolonthinae, Adoretini, Anomalini, Dynastinae, and Cetoniinae) followed the rapid diversification of the angiosperms during the Late Cretaceous – Early Palaeogene. Unlike in dung beetles, considered a model group for comparative studies of niche partitioning and functional structure [12]–[17], food resources of the phytophagous pleurosticts are less patchy in distribution and less ephemeral. While the food resources of the saprophagous ancestors of the pleurostict scarabs [11] were restricted to a relatively limited two-dimensional stratum (upper soil layers), with the rise of angiosperms a vast food space became available [18]. It is assumed that this new third dimension of food availability generally reduced competition among herbivores [18], [19] and the exploitation of different parts of the plants, like roots, stems, leaves, and florescences [3], [11] provided further possibilities for avoiding competition. The presence of various aggregation mechanisms (volatiles, pheromones) for host location and/or mate finding [3], [20], [21] and a highly complex chemical ecology [22]–[25] seem to support this hypothesis.

If competition for food resources triggered morphospace diversification and assemblage structure in pleurosticts, we would, as in the closely related dung beetles, observe an increased divergence of morphospace among similar feeding types. Alternatively, if we would observe less or no divergence in morphospace between similarly feeding lineages, we would expect little or no directed selection on morphological traits. However, further environmental pressures may also cause divergence.

While actual inter-specific competition is difficult to measure and needs to be explored at the assemblage level [17], competition in the past that no longer exists due to partitioning of the species niche or extinction of less competitive species (‘ghost of competition past’, [26]) may be reflected in the morphospace. However, phylogenetic lineages will differ in morphospace if their common ancestors did, because members of a lineage share a greater similarity in their morphology as a result of the lingering legacy of a common ancestor [27]–[30]. Competition that led to niche partitioning in the ancestors of extant lineages is therefore also visible at a phylogenetic level. However, under the hypothesis of reduced competition we might also encounter divergence between different feeding types (herbivorous, floricolous, etc.), despite their spatial avoidance of competition, due to subsequent adaptation to the life style in relation to the use of a new food resource.

Here we used a multivariate analysis of body length measurements of external body morphology that was linked to a phylogenetic hypothesis of the group [31] to investigate the evolution of scarab morphospace. Our ecomorphological approach followed Wainwright and Reilly [32] assuming that body size and allometric shape variation reflect differences in the species ecology and behavior [33]–[37]. Additionally, we explored the presence of evolutionary key innovations that were possibly linked with quantitative traits of body shape and that might have promoted the diversification of certain lineages [38]. We explored the morphospace divergence in a twofold approach: 1) Searching for simple phenetic divergence at a nested level and detecting which traits contribute most to the observed divergences. I.e. searching simply for differences between the different feeding types, major lineages, and sister clades. Our null assumption was “no divergence – no competition” such that among species of the same feeding type that do exhibit no or very little divergence in morphospace no competition occurs. 2) Exploring morphospace divergence in relation to molecular rates of evolution: Through the link with the molecular branch lengths we were able to infer directed selection that is linked with significant divergence of body morphospace at any phylogenetic level. For traits under neutral evolution and therefore stochastic drift, rates of morphological change are correlated with those of molecular evolution. Observed divergence and uncorrelated rates among similar feeding types would provide insight, whether (and what kind of) directed selection (likely as result of competition avoidance) had an impact on pleurostict morphospace divergence, which would allow to identify key factors of the successful scarab diversification.

## Materials and Methods

### Ethics statement

We obtained permission from the Zoological Research Museum A. Koenig, Bonn (ZFMK) to access, loan and dissect the material in the collections.

### Taxon sampling and morphometric measurements

Based on the phylogenetic analysis of Ahrens and Vogler [31], we sampled a single specimen of 182 species of all principal lineages of phytophagous Scarabaeidae from Neotropical, Palearctic, Afrotropical, Oriental, and Australasian regions (see Table S1). Vouchers are deposited in the collection of the Zoological Research Museum A. Koenig, Bonn (ZFMK).

**Figure 1:**
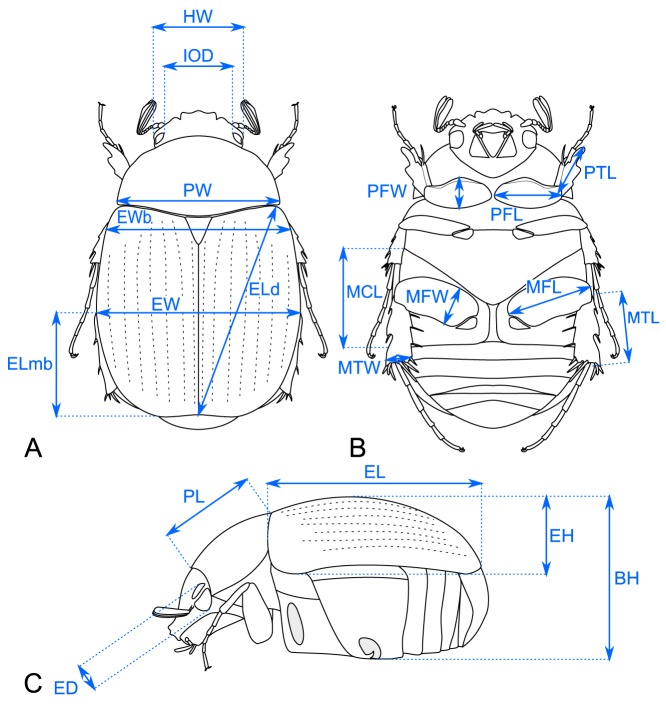
Illustration of the measured traits. Schematic drawings of a Sericini beetle in (A) dorsal, (B) ventral, and (C) lateral aspect. **Body:** ***BH -*** maximal body height, ***EH -*** maximal elytra height, ***EL -*** maximal elytra length, ***Eld -*** maximal diagonal elytra length, ***Elmb -*** length from maximal body width to elytral apex, ***EW -*** maximal elytra width, ***Ewb -*** elytral width at middle of scutellum, ***PL -*** maximal pronotum length, ***PW -*** maximal pronotum width; **Head:** ***ED -*** maximal eye diameter, ***HW -*** maximal head with including eyes, ***IOD -*** minimal interocular distance (dorsal view); **Legs:** ***MCL -*** maximal length of metacoxa, ***MFL -*** maximal length of metafemur, ***MFW -*** maximal width of metafemur, ***MTL -*** maximal length of metatibia, ***MTW -*** maximal width of metatibia, ***PFL -*** maximal length of profemur, ***PFW -*** maximal width of profemur, ***PTL -*** maximal length of protibia.

Twenty linear distance measurements were performed on adult beetles to capture the complexity of body shape (Figure 1A–C). Characters subjected to a strong sexual dimorphism were not used because female and male specimens were included in the molecular phylogenetic analysis [31]. The measurements were taken directly (where possible) from the sequenced voucher specimens of Ahrens & Vogler [31] with the help of an ocular grid on a Zeiss SM20 Stereomicroscope, and values were converted to millimeters for the different magnifications. Measurements were taken in such a way that the endpoints were in focus. In order to reduce the variance introduced by several sources of subjective measurement errors [39], the measurements of all specimens were repeated 5 times and subsequent analyses were conducted with the means of the measured values.

### Analysis of morphospace

Analyses of morphospace were implemented based on the Bayesian phylogenetic tree [31] on the preferred alignment as a backbone. Morphospace was explored for the complete data set of all specimens and for five subsets that compare major sister clades. Comparisons between sister clades with low support values are omitted except those with relationships that are also well established in traditional morphology-based systematics. All calculations for the analysis of morphospace were made within the R statistics environment version 2.15 [40] unless otherwise stated. Obtained linear measurements were log_10_-transformed to render more linear relations among variables and to obtain a similar dimension of variance [41], [42]. Generally, the major component of variance in morphometric data sets of biological specimens is explained through size [43]–[45]. To avoid a strong bias of size over variation of shape, we employed approaches that separate size from shape information. In landmark-based geometric morphometrics, this is achieved using “two point registration” methods [46], [47], but for linear measurements there is still some debate regarding how to perform this separation [47], [48]. Here, we employed the Burnaby Back Projection Method (BBPM) [43] by projecting the log-transformed data on the isometric size vector and returning it to the original coordinate system [48], [49] as implemented in an R-code provided by Blankers et al. [49]. This method has the advantage of deriving a composite measure of size from all traits and considering shape as the projection onto the orthogonal space of this isometric vector. Data treated in this manner are subsequently referred to as size-corrected data (set). Correcting the data for size can strongly affect the results depending on the method used and must be considered well. Therefore, we compared the results from the BBPM with shape data derived from a linear regression (residuals) against overall body length [50], [51] which was chosen to be representative of the beetles' body size. Because a high error is introduced to the total body length measure through the motility of the prothorax against the pterothorax, a proxy was used by calculating the logarithm of the sum of pronotal and elytral length (log(PL+EL)). The impact of size (percentage of variation that is explained by size alone) was assumed to be represented by the percentage of variation explained by the first principal component of the uncorrected data set.

Patterns of morphometric covariation were analyzed with standard principal component analysis (PCAs; [52], [53]) on uncorrected and size-corrected data. Results were visualized with the help of the ade4 package [54]. Additionally, the molecular phylogeny was projected onto the morphospace explained by PCs 1 and 2 using the function *phylomorphospace* in the R package phytools [55]. The program therefore estimates the positions of the ancestral nodes using a maximum likelihood approach.

Statistical evaluation of group differentiation in morphospace was done by MANOVA and linear discriminant analysis (LDA). To avoid confusion through noise introduced from measurement errors or minor unspecific variation [56]–[58], we only used the principal components that explained 95% of total variation. Non-parametric MANOVA [59] was performed for the complete data set and each sister clade subset in PAST 2.17 [60] to test for significant differentiation between lineages. Sequential Bonferroni [61] correction was applied. LDA was conducted on the same groupings to evaluate group discrimination by the reassignment probabilities [62] which were evaluated by leave-one-out cross-validation using the MASS-package [63] in R. Lineages represented only by a single species, i.e. Ablaberini, were included in the PCA but had to be excluded from LDA and MANOVA.

### Feeding habits and morphospace

Inference of the potential influence of the food resource on morphospace variation was done by mapping feeding habits of each species onto morphospace. Details on feeding behavior were taken from the literature and were complemented with personal observations (Table S1). Coprophagous (COP) and saprophagous (SAP) species were represented by Aphodiinae/Scarabaeinae and Hybosoridae, respectively. Anthophilous (ANT) species exclusively forage on flowers, feeding on pollen and nectar, whereas herbivorous species (HERB) devour various plant materials, including foliage, twigs, and petals. Dynastinae species examined here are sap/fluid utilizers (SFU) feeding under ground on stems or roots in order to gain access to fluids from the wounds [3]. Adults of *Pachypus* do not feed (NF).

A correlation analysis between morphospace and feeding types was performed employing phylogenetic generalized least squares regression in the package caper using the *pgls* function [64]. The assigned feeding types were used as independent variables and (standard) principal components explaining 95% of cumulative variation as dependent variables representing the morphospace. To improve the fit of the data to the tree, Pagel's [65] branch length transformation variable λ (internal branch lengths are multiplied with λ) was set to be estimated by maximum likelihood. κ (each branch length is raised to the power κ, [65]) and δ (the node heights are raised to the power delta, [65]) were set at 1.

A possible correlation between molecular and morphological distances between the specimens was estimated by Mantel-tests, performing 10,000 permutations of Pearson correlations with the R package vegan [66]. The analysis was made for size-corrected data for all members of each feeding type separately.

### Detecting driven selection and key innovations

Reduced correlation between molecular and multivariate morphometric distances is likely to indicate decoupling of molecular and morphological rates of evolution, with accelerated or decelerated rates of evolution in either of the traits, i.e. directed selection on morphospace evolution. Therefore, Mantel-tests with Pearson correlation were performed on distance matrices of patristic distances (calculated with the *cophenetic* function in the R-package ape [67] from the molecular tree [31]) and Euclidean distances of the respective morphological data sets. To infer individual traits that underlay directed selection, i.e. that deviate from Brownian Motion, the descriptive K statistic of Blomberg et al. [68] was calculated for every trait over the complete size-corrected data set using the R package phytools [55], [69]. A K value greater than one implies that close relatives are more similar than expected under Brownian motion evolution [68].

Branches in the phylogeny, where the molecular and the morphological distances between nodes deviate from each other, were detected by projecting both the uncorrected and the size-corrected data set on the constrained topology of the phylogenetic tree [70], [71]. The branch lengths were inferred with the *optim.phylo.ls-*function from the phytools package [55] using Euclidean distance matrices of the respective data sets. Negative branches were set to zero. For both the size-corrected and the uncorrected data set, ratios of morphological and molecular branch lengths were calculated for each branch. Values above and below the 95% confidence interval of the ratios were considered as significantly different in their branch lengths, i.e. indicating an extraordinary decoupling of molecular and morphological rates and consequently directed selection at the respective ‘outlier’ branch. Because the lengths of internal branches and tips often largely differed, they were evaluated separately. (cf. Figure S4)

Additionally, we calculated standardized phylogenetic independent contrasts [72]–[74] in order to compare evolutionary rates of morphospace divergence between clades. For this objective, we used the multivariate approach introduced by McPeek et al. [75] and applied it to both data sets. The method of McPeek et al. [75] was implemented in R and the script is provided in Script S1. The ultrametric tree necessary for this approach was calculated based on the preferred alignment (2513 bp) of Ahrens and Vogler [31] using PathD8 [76], with the root of an arbitrary age of one. Ancestral linear size measurements of traits possibly linked with presumptive key innovations were reconstructed with the function *fastAnc* in phytools [55] using a Maximum Likelihood approach.

## Results

**Figure 2:**
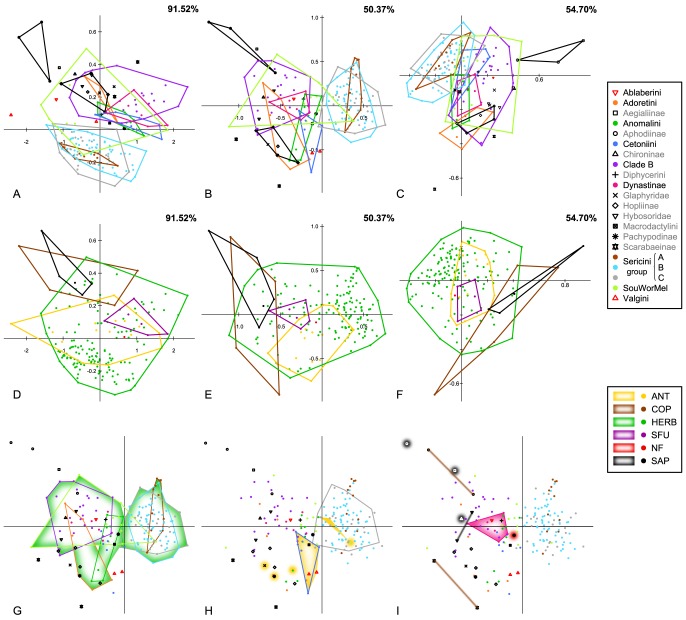
Patterns of morphospace covariation between major phylogenetic lineages and feeding types. Scatterplots of the principal component scores from the analysis of the complete sampling of (A, D) the uncorrected and the size-corrected data sets from (B, E) the Burnaby Back Projection Method (BBPM) and (C, F) the linear regression method with (A–C) major phylogenetic lineages and (D–F) feeding types projected on it (ANT  =  anthophilous, COP  =  coprophagous, HERB  =  herbivorous, SFU  =  sap/fluid utilizers, NF  =  not feeding, SAP  =  saprophagous). The percentage of variance explained by principal component 1 and 2 is given in each upper right corner. Taxa with more than 2 members are surrounded by a similarly colored hull. (G–I) Morphospace divergence within the feeding types projected on scatterplots of the principal component scores from size corrected data (BBPM): (G) Herbivores, (H) anthophilous, and (I) the remaining feeding types. Dots are color-coded in the molecular phylogeny (Figure 3A) for phylogenetic lineages. x-axis: PC1, y-axis: PC2.

**Figure 3:**
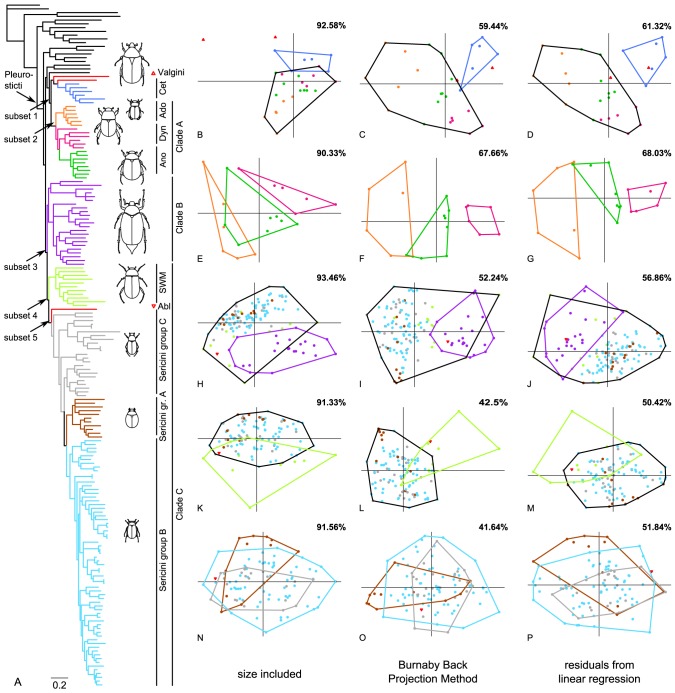
Backbone phylogeny and morphospace covariation between sister clade subsets of the complete sampling. (A) Phylogenetic tree of major scarab lineages from the Bayesian analysis of Ahrens and Vogler [31]. Scatterplots of the principal component scores for the uncorrected and the size-corrected data sets: (B–D) Cetoniini (Cet) + Valgini and Adoretini (Ado) + Anomalini (Ano) + Dynastinae (Dyn), (E–G) Adoretini, Anomalini, and Dynastinae, (H–J) Clade B and Southern World Melolonthinae (SWM) + Ablaberini (Abl) + Sericini, (K–M) Southern World Melolonthinae and Ablaberini + Sericini, and (N–P) Ablaberini and Sericini subgroups. The groups are color-coded in the phylogeny and the scatterplots. The percentage of variance explained by principal component 1 and 2 is given in the top right corner. Groups with more than 2 members are surrounded by a similarly colored hull. Black hulls border sister lineages for illustration of divergence. x-axis: PC1, y-axis: PC2.

Results for the two methods for removing isometric size from the data, the Burnaby Back Projection Method (BBPM) and linear regression against a size metric, were quite similar (Figures 2, 3). Therefore most results for the latter method are presented in the supplement information only (Tables S10–S12, Figure S5) and were compared concisely to those of BBPM in the discussion.

### Feeding habits and morphospace

The phylogenetic generalized least squares analysis did not recover any significant correlation between the feeding types and the morphospace. The r^2^-values of the regression were low for size-corrected and uncorrected data (adjusted r^2^ = 0.031 and 0.025, respectively). Plots of the PCA-scores of PCs 1 and 2 of the complete data set analysis were very similar for the size-corrected and the uncorrected data set (Figure 2D–F), showing a large overlap of all phytophagous groups (anthophilous, herbivorous, and sap/fluid feeders). Non-feeders showed no separation from these groups. Coprophagous and saprophagous feeders appeared somewhat divergent from the phytophagous groups, although an overlap in particular with the herbivores, which occupied a very vast morphospace, was also present. Separate clusters became evident for all feeding types except juicy feeders when projecting feeding types on the morphospace of major lineages (Figure 2G–I).

Significant correlations between molecular and morphological distances could not be found within any of the feeding types.

### Morphospace divergence of phylogenetic lineages

The impact of size (the variation of uncorrected data explained by PC1 alone), was high among the complete sampling and all subsets (82.4% to 89.5%, Table S9). A large overlap of most lineages was visible from the scatterplots of the scores of principal components 1 and 2 for both the size-corrected and the uncorrected data set (Figure 2A–B). However, in morphospace Aphodiinae and Sericini were quite separate from the other lineages in both data sets. Despite the large overlap of the lineages in the PCA scatterplots, the MANOVAs showed generally significant differentiations between the major lineages within the size-corrected as well as the uncorrected data set (size-corrected data set: F = 13.42, p<0.0001; uncorrected data set: F = 8.49, p<0.0001). However, only 38 of the 105 pair-wise comparisons yielded significant results for the size-corrected data and only 14 were significant for the uncorrected data (Table 1). Most of the significant results involved one of the three Sericini subgroups. The lineages of Adoretini, Glaphyridae, Hopliinae, Hybosoridae, Scarabaeinae, and Southern World Melolonthinae were distinguished only by the size-corrected data set from at least one other clade. Valgini did not yield significant results. MANOVA of the uncorrected data gained higher F-values in comparison to those from the analysis of the size-corrected data set (6 out of 14 with higher F-values) for lineages that were represented by many large species (EL+PL>15 mm), such as Cetoniini, Clade B, and Dynastinae.

**Table 1:** F-values from non-parametric MANOVA of the complete sampling (excluding singletons) regarding 95% of total variation.

	Adoretini	Anomalini	Aphodiinae	Cetoniini	Clade B	Dynastinae	Glaphyridae	Hopliinae	Hybosoridae	Scarabaeinae	Sericini A	Sericini B	Sericini C	SWM^1^	Valgini
Adoretini		4.96*	9.45*	12.34*	***6.80***	12.64*	3.71*	3.80*	3.49*	6.34*	***16.90***	***24.50***	***21.45***	***5.88***	6.42*
Anomalini	5.22*		12.78*	6.07*	***6.04***	***5.47***	3.61*	4.55*	2.64*	6.96*	**10.22**	***13.50***	**10.46**	1.96	4.13*
Aphodiinae	39.30*	47.75*		11.95*	**8.43**	11.87*	6.02	12.79*	3.10	4.96	16.32*	***31.88***	***25.77***	6.86*	7.28
Cetoniini	7.73*	1.29	33.83*		***13.97***	9.67*	4.07*	8.71*	5.72*	6.16*	**12.61**	***20.71***	**14.15**	**4.17**	3.55*
Clade B	3.96	0.88	***21.07***	0.55		***9.91***	3.75*	**5.97**	1.95	8.66*	***24.02***	***57.48***	***36.40***	**5.15**	8.58*
Dynastinae	13.83*	3.12	49.12*	0.45	0.98		9.10*	13.32*	1.91	7.90*	**12.24**	***23.52***	***18.27***	**5.24**	5.12*
Glaphyridae	0.39	1.41	24.58	2.10	1.01	4.37		1.95	6.58	3.71	10.24*	***12.05***	10.05*	2.47	6.17
Hopliinae	1.30	8.40*	17.05*	9.73*	6.21*	15.44*	1.18		5.63*	6.42*	**19.17**	***24.37***	***20.56***	5.06*	7.57*
Hybosoridae	2.87	6.29*	13.29	5.92	3.02	9.76*	5.89	0.56		2.99	7.88*	***11.11***	9.72*	2.10	4.57
Scarabaeinae	5.13	0.81	30.33	0.47	0.38	1.13	1.93	5.16	8.10		14.28*	***19.66***	15.85*	4.87*	2.44
Sericini A	8.01*	***20.27***	17.92*	***22.65***	**19.12**	***30.91***	4.81*	3.77*	1.63	11.26*		***5.32***	4.51*	***6.47***	6.59*
Sericini B	2.58	9.99*	**24.01**	**16.18**	**32.11**	**21.02**	1.56	2.64	1.51	5.19*	3.90*		1.76	***11.46***	9.54*
Sericini C	5.51*	***18.89***	**17.44**	***23.88***	**30.56**	**30.92**	3.17	2.51	1.24	8.68*	0.47	4.64*		***7.24***	7.56*
SWM^1^	0.43	3.28	5.76*	5.05*	7.61*	6.50*	0.35	0.24	0.14	1.47	1.10	1.19	1.22		3.37*
Valgini	4.83	9.63*	0.82	8.25	8.61*	10.72*	1.59	2.09	0.69	2.69	2.82	7.14*	3.56	1.57	

The values for the size-corrected data set are shown in the upper triangle, those for the uncorrected in the lower one. Significant differences (p<0.05) are highlighted in bold. Higher F-values for the same significant pairings are printed in italics in the respective triangle.

* Significant without sequential Bonferroni correction.

1 Southern World Melolonthinae.

The comparison of sister lineages (Figure 3B–P) was not influenced by the interference of variation from other lineages. While subsets 1 and 2 showed an improved differentiation compared to the analysis of the complete sampling, the patterns of morphospace-distribution changed only marginally for subsets 3, 4 and 5. However, the comparisons of sister lineages generally showed more specific information about which part of body shape (PC vectors of the subset) represent the morphological divergence of sister clades. Size correction improved the outcome only slightly in these comparisons (Figure 3).

The two major lineages of subset 1 (Cetoniinae vs. Clade A; Figure 3B–C) are well differentiated for the uncorrected and the size-corrected data set although a slight overlap was present. These results were congruent with the results of the MANOVAs of subset 1 where the analysis of the size-corrected data set resulted in a nearly 8-fold higher F-value (Table 2), while F-values of uncorrected data were not significant. LDA incorrectly reassigned five specimens with the uncorrected data set (85.71% correctly reassigned, Figure S1A), but only three specimens with the size-corrected data set (89.29% correctly reassigned, Figure S1B). *Microvalgus* was only correctly assigned to Valgini with the size-corrected data set.

**Table 2:** F-values from non-parametric MANOVA (Anderson 2001) of each subset (ss1–ss5, excluding singletons) regarding 95% of total variation.

**Subset 1**	Cetoniinae	Clade A	
Cetoniinae		**6.79**	
Clade A	0.87		
**Subset 2**	Adoretini	Anomalini	Dynastinae
Adoretini		**5.13**	**12.96**
Anomalini	**0.02**		**5.61**
Dynastinae	**0.00**	0.10	
**Subset 3**	Clade B	Clade C	
Clade B		**47.90**	
Clade C	**41.85**		
**Subset 4**	Sericini	SWM^1^	
Sericini		**11.02**	
SWM^1^	1.08		
**Subset 5**	Sericini A	Sericini B	Sericini C
Sericini A		**5.29**	**4.83**
Sericini B	3.82		**2.11**
Sericini C	0.56	4.58	

The values for the size-corrected data set are shown in the upper triangle, those for the uncorrected in the lower one. Significant differences (p<0.05) are highlighted in bold.

1 Southern World Melolonthinae.

For subset 2, both data sets reveal a differentiation between groups, although a slight overlap was present in the uncorrected data set (Figure 3E–G). Anomalini take an intermediate position in morphospace between Adoretini and Dynastinae. MANOVA showed only slight separation for the uncorrected data compared to the size-corrected data (Table 2). LDA correctly reassigned 90% of the specimens to the respective groups for the size-corrected data set, but still 80% for the uncorrected data set (Figure S3C–D).

Scatterplots of the first two PC axes of subset 3 (comprising all ‘Melolonthinae’) show a large overlap of Clade B and Clade C in the size-corrected and the uncorrected data set. This is mainly caused by the Southern World Melolonthinae that widely ‘invade’ the morphospace of Clade B (Figure 3H–J; green dots). MANOVA, which considers multiple PCA dimensions of the morphospace, suggests a distinct separation of the two sister lineages. The F-value was slightly higher for the size-corrected data set (Table 2) what was consistent with the reassignment probabilities from LDA (correctly reassigned for uncorrected data: 95.52%, for size-corrected data: 96.27%). Although the number of specimens correctly reassigned by the discriminant function was nearly equal in subset 3, the number of specimens with reassignment probabilities over 95% decreased from 96% to 94% (Figure S1E–F). In contrast to all others, for this data subset the membership to the predefined groups could be recovered more unambiguously with the uncorrected data.

For data subset 4, the correction for size resulted in no marked differences in patterns of the specimen-distribution in morphospace, and Ablaberini + Sericini and Southern World Melolonthinae showed a considerable overlap in morphospace (Figure 3K–M). MANOVA's F-values resulting from the size-corrected data set are about 10-fold higher, and those of uncorrected data were not significant (Table 2). LDA on the size-corrected data set correctly reassigned 92.79% of the specimens (63.64% of Southern World Melolonthinae) whereas 90.99% (only 18.18% of SWM) were correctly reassigned for the uncorrected data set.

Subset 5 (Sericini) was sampled more in detail [31] and was further subdivided into three groups (Figure 3N–P): Group A (subtribe Trochalina) is monophyletic and partly characterized by a more or less spherical body shape (genus *Trochalus*), group B (subtribe Sericina) is the monophyletic sister clade to the Trochalina and is characterized by a more oval body shape, while group C represents the paraphyletic remainder of Sericini basal to group A+B. Based on our measurements, Sericini subgroups differed only slightly in morphospace (Figure 3N–P). MANOVA on the size-corrected data set supported these results suggesting a differentiation of group A (Trochalina) from both other subgroups of Sericini (Table 2). MANOVA on uncorrected data revealed no significant differentiation. The reassignment of specimens to the predefined groups by the discriminant function was improved through the size-correction of the data set (correctly reassigned for uncorrected data set: 69.0%, for size-corrected data set: 75.0%; Figure S1I–J).

**Figure 4:**
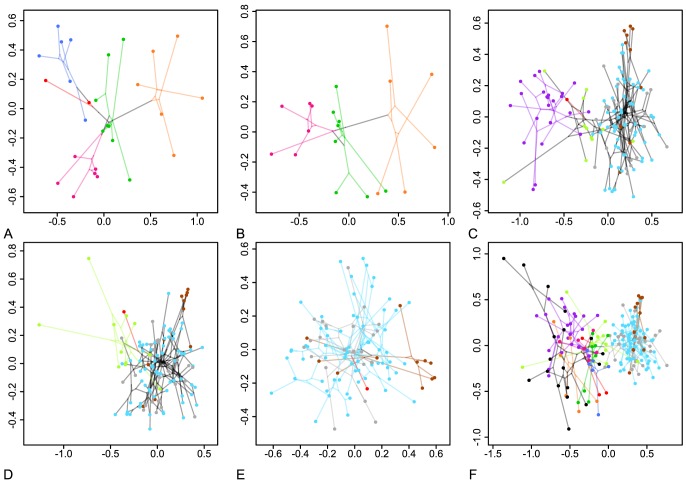
Lineage diversifications in morphospace. Phylomorphospace projections of the molecular phylogenetic tree [31] for the sister clade subsets 1–5 (A–E) and the complete data set (F) showing the first two PC axes of the size-corrected (BBPM) data set.

The projection of the phylogenetic tree onto PCs 1 and 2 revealed the divergence within the subsets and clades (Figure 4). Analysis of subsets 1 and 2 revealed a shift in morphospace of the ancestors of Anomalini, Adoretini, Cetoniinae, and Dynastinae, with subsequent diversification within the respective morphospace units. A similar result was observed for the genus *Trochalus* (Figure 4E, lower half, brown dots).

### Which traits shape the morphospace divergence?

Because the directions of morphospace divergences between the lineages were much more evident from the size-corrected data, we used these to investigate trait behavior in the context of the measured divergence. PCA vector loadings allowed us to draw conclusions about the contribution of traits to the morphological divergence of the lineages (Tables S2–S7, Figure S2). Scarab morphospace (PC axes 1 and 2) inferred from the complete data set (Figure 2B, Table S2) was principally influenced by traits of limb length (PTL, MTL, PFL, MFL) and elytral height (EH). The strongest influence in total was metacoxal length (MCL).

Observed principal components (PCs) of variation from sister clade comparisons (subsets 1–5) were not influenced by the interference of variation with other lineages, and thus we were able to detect morphological divergence linked with the divergence of sister lineages. In comparison to its sister clade A (including Adoretini, Anomalini, and Dynastinae), Cetoniinae were mainly characterized by traits that are equivalent for a relatively smaller head (HW, IOD), smaller eyes (ED), a longer pronotum (PL), and a dorso-ventrally flattened body (EH; Table S3, Figure S2B–C). Adoretini were found to have a wider head and larger eyes (HW, IOD, ED), a shorter pronotum (PL) compared to Dynastinae (Table S4, Figure S2E–F). Dynastinae had shorter and stouter extremities. Anomalini had an intermediate position in morphospace between Adoretini and Dynastinae. Within Melolonthinae (subset 3) there was an overwhelming influence of metacoxal length (MCL, Table S5). Also the width of hind limbs contributed to the differentiation of the sister clades. The influence of MCL was distinctly reduced when Clade B was excluded (subset 4, Table S6, Figure S2K–L). However, the major part of Sericini was still found to be divergent in longer metacoxa and broader hind limbs in general. Within Sericini (subset 5), specimens with a more spherical appearance (higher values for EW, EWb, PW, EH, and BH but also PL; Table S7) were located in the left side of the plot (Figures 3O, S2N–O), whereas more elongate specimens were located in the right side of the plot. Therefore, a significant shape divergence must have occurred within Sericini group A with *Trochalus* appearing on the extreme left side of total variation along the x-axis while *Allokotarsa, Idaeserica*, and *Ablaberoides* are more centered in the plot.

**Figure 5:**
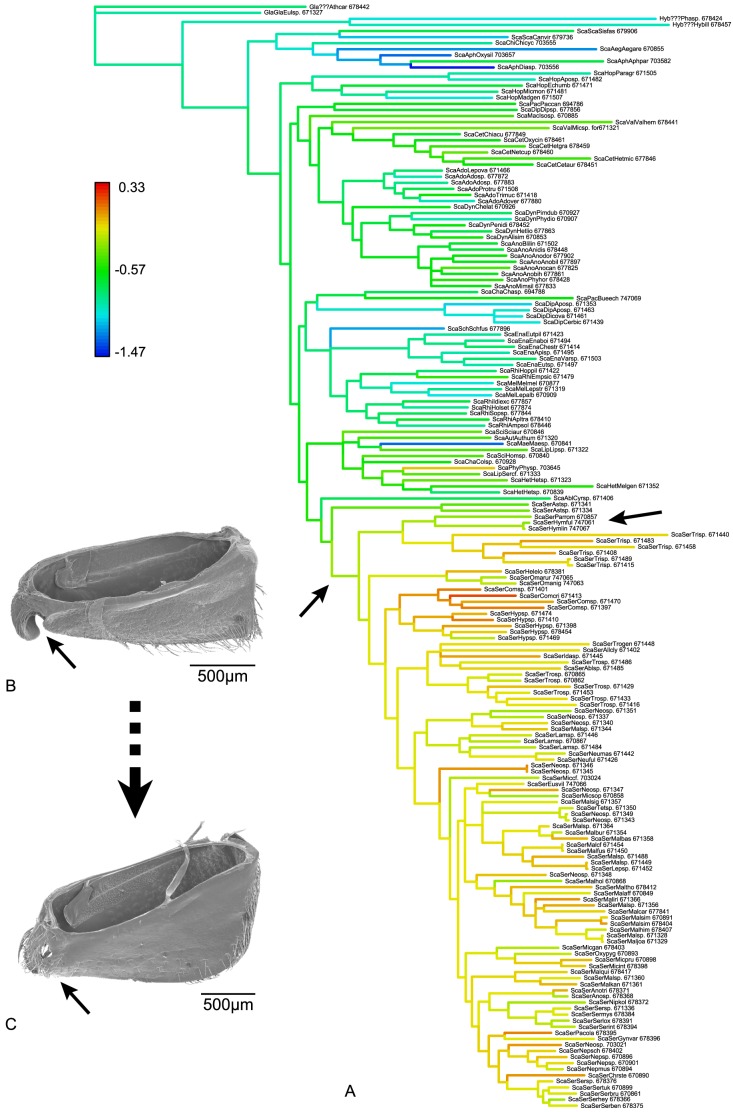
Correlated evolution of metacoxal length and the secondary metacoxal ostium. (A) Reconstruction of relative metacoxal length in ancestral nodes of the molecular phylogeny [31]. The left hand arrow shows the internal branch where ancestral relative metacoxal length strongly increases and where the secondary ostium of metacoxa is closed by the medial apophysis [77]. The right hand arrow points to the clade of *Hymenoplia* and *Paratriodonta* (see text for explanation). (B) *Chasmatopterus* spec., metacoxa, dorsal view: secondary ostium open (arrow). (C) *Hymenoplia castilliana*, metacoxa, dorsal view: secondary ostium closed (arrow). The numbers in the legend correspond to the size-corrected values of metacoxal length.

The inference of the influence of the measured traits on scarab morphospace (Table S2) is complemented by the estimated phylogenetic signal (descriptive K-statistics, Table S2, [68]) of every trait from the size-corrected data set. All traits except MCL have K-values below 1, indicating that they tend to exhibit a weaker signal than expected under Brownian motion model [68]. The K-value found for the metacoxal length (MCL; K = 3.21) was with distance the highest value, indicating a higher conservatism for this trait, with close relatives being more similar than expected under Brownian motion evolution and though possibly indicating directed selection. However, the absolute amount of K was highly influenced by the biased sampling towards Sericini in our study. In fact, if we simulated a stepwise decreased amount of Sericini by pruning species of this lineage from the tree and the morphometric data set, the K-value went below 1 (with 3 sampled species of Sericini, Figure S3C). The K-value for MCL was, however, always the highest or second highest value in total. A subsequent maximum likelihood reconstruction of the size-corrected MCL (Figure 5A) on the tree revealed a strong shift of MCL's relative length at branches of ancestral Sericini (Figure 5A, left hand arrow).

### Morphological vs. molecular rates of evolution

The Mantel tests between molecular and morphological distance matrices were significant only for the size-corrected data of the complete sampling. Correlation was low (r = 0.11, p = 0.01).

**Figure 6:**
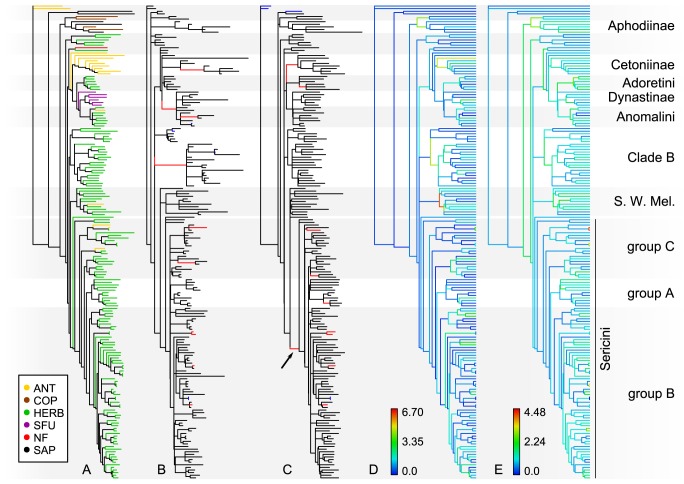
Morphological divergence in multivariate space and rates of morphological divergence. (A) Molecular phylogenetic tree [31], trees with optimized branch lengths by (B) the uncorrected and (C) the size-corrected data set (BBPM), and rates of morphological divergence (multivariate standardized phylogenetic independent contrasts) for (D) the uncorrected and (E) the size-corrected data set mapped on the ultrametric phylogenetic tree showing relative divergence times. The tips of the molecular tree (A) are color-coded for feeding habits (ANT  =  anthophilous, COP  =  coprophagous, HERB  =  herbivorous, SFU  =  sap/fluid utilizers, NF  =  not feeding, SAP  =  saprophagous). Branches in (B) and (C) with significantly lower (blue) and higher (red) morphological rates of evolution are colored respectively. Background shading indicates clade affiliation.

Optimization of morphospace variation onto the phylogenetic tree provided a better measure of the relative morphological divergence of phylogenetic lineages (Figure 6B–C), allowing a more general assessment of morphological change at diverse phylogenetic levels, especially when extraordinary rate decoupling was identified through rate ratio outliers (Figure 5B,C, Figure S4). Given that variation in the uncorrected data set was mainly induced by size-differences of the species, long branches in the respective tree should be mainly attributed to change in size of the hypothetical ancestor of the group. Long branches that result from the size-corrected data set, however, indicated a higher rate of change in shape. Internal branches were of highest interest for the inference of morphological lineage divergence because they represented change in morphospace common to a whole clade. Terminal optimized branches, however, were generally longer than internal ones, suggesting that only a very few morphospace shifts exceeded interspecific variation of extant taxa within selected lineages.

Only a few specimens within Sericini group C and none of the internal branches coincidently showed significant branch length differences, thus supporting increased morphological change and presumed directed selection (Figure 6B–C; in both, the size-corrected and the uncorrected data set). Most lineages either diverged primarily in size (i.e. the uncorrected data set) or shape (i.e. in the size-corrected data). Cetoniinae were divergent from Adoretini + Anomalini + Dynastinae in shape (Figure 6C), whereas the clade of Anomalini + Dynastinae clearly showed a common divergence in size from Adoretini (Figure 6B). Clade B was found to have common (although little) divergence in shape, whereas two subordinate lineages strongly differed in size (Figure 6B–C). Rates of evolution were significantly different for shape and the molecular markers in the branch leading to the most recent common ancestor of Sericini. The genus *Trochalus* showed conspicuous divergence in shape within the Sericini group A (Figure 6C).

Phylogenetic independent contrasts revealed, in part, strong changes of rate of morphological evolution for both the uncorrected and the size-corrected data (i.e. shape, Figure 6D–E). Among larger clades, we found strong rate shifts among both data sets between Aphodiinae and Scarabaeinae, within the Southern World Melolonthinae, but also within Clade B (slightly more distinct pattern in uncorrected data, Figure 6D). For several clades with low contrasts for uncorrected data we found elevated contrasts for the size-corrected data. For the size-corrected data, deeper branches (e.g. between saprophagous and coprophagous Scarabaeidae and Pleurosticti) were found to exhibit major morphological change whereas the uncorrected data set revealed stronger change and frequently accelerated rates of morphological divergence on more recent time scales (Figure 6B–E). Several closely related taxa (i.e. terminal species pairings), especially within the densely sampled Sericini, exhibit higher rates of divergent evolution.

## Discussion

### Morphospace divergence in the light of feeding habits

Because morphological traits are an important expression of the species niche, a partitioning of the niche as a consequence of interspecific competition between coexisting species may lead to divergence in ecomorphospace [10], [29]. A directed selective force on external morphology is likely to alter the rate of morphological evolution. Rates are then unlinked from the Brownian Motion model [27], [77] which is assumed by our approach in the molecular phylogenetic framework based on 16S, 28S and Cox1 [31], [78]. While actual inter-specific competition is difficult to measure and needs to be explored at the assemblage level, competition in the past that led to a partitioning of the species niche or extinction of less competitive species (‘ghost of competition past’, [26]) may be reflected in morphospace; phylogenetic lineages will differ in morphospace based on historical constraints of their common ancestors and because members of a lineage share a greater similarity in their morphology as a result of the lingering legacy of a common ancestor [27]–[30]. Competition that led to niche partitioning in the ancestors of extant lineages is, to some degree, also visible at the phylogenetic level. Evolution of dung beetles, the nearest relatives of Pleurosticti, was markedly influenced by strong competition for their food resource: the resulting resource partitioning led to divergence in morphospace which presumably triggered the diversification of the dung beetles [10]. To assume an analogous situation for pleurosticts would be hard to prove since competition between adults has not been shown yet in literature. We therefore assumed as null hypothesis the reverse: if no divergence in morphospace is found, it should be concluded that there is no resource competition among species of the same feeding type and that there is any other selective pressure. However, the opposite does not necessarily mean that competition is the cause of divergence.

For example, different feeding type assemblages of adult pleurosticts are generally spatially separated (e.g. flowers, leaves, wood), but different locomotion behavior may be required besides other adaptations. Body traits such as legs and parts of the flight apparatus are therefore also likely to cause divergence in morphospace, as found for Dynastinae and rose chafers.

Results indicate directed selection in pleurostict chafers that explains morphological expansion of feeding types. A vast portion of the wide overlap between the different groups of feeding behavior can be explained by convergence of feeding behavior for most of the feeding types (Figure 2G–I). In particular, repeated shifts from herbivory to anthophagy (e.g. in Sericini, Hopliini, Southern World Melolonthines; Figure 6), linked with the rise of the angiosperms, offered large amounts of new nutritious resources.

Also within one feeding type, rates of morphological evolution departed from Brownian motion. Besides multiple rate shifts (Figures 6D–E), we observed an indication of decoupling of morphological rates of evolution from Brownian motion in herbivores (Table 3) that favored a hypothesis of directed selection on morphospace. The general difficulty to detect missing correlation of morphological and molecular rates could not be overcome even by better sampling, as results of well-sampled herbivore chafers show. Multiple shifts of rates of morphological evolution suggested that directed selection on morphospace took place within herbivores. Especially one trait, metacoxal length, caused distinct divergence of the herbivores morphospace (Figure S6). This would reject at least in part the idea that the vast Angiosperm feeding resource available to phytophagous scarabs would result in lacking divergence (i.e. stochastic morphological drift in morphospace dimensions).

**Table 3:** Correlation between molecular and morphometric distance-matrices for specimens within one feeding type and the complete sampling.

	sample size	r	p
ANT^1^	16	−0.01	0.51
COP^^2^^	4	−0.01	0.51
HERB^^3^^	150	0.04	0.15
SFU^4^	6	−0.06	0.52
SAP^5^	5	−0.61	0.97
complete sampling	182	0.11	0.01

Sample size, coefficient of determination, and p-values from Mantel-tests are given.

1 anthophilous;

2 coprophagous;

3 herbivorous;

4 sap/fluid utilizers;

5 saprophagous.

### Morphospace divergence of phylogenetic lineages

When discussing lineage divergence and all other topology related issues, uncertainty of the phylogenetic hypothesis is a major bias. Therefore, the majority of our analyses focused on well-supported clades, which were retrieved also in other studies [79]–[81]. While major phylogenetic lineages of Scarabaeidae generally showed large overlap for principal components 1 and 2 in both data sets (Figure 2A–B), significant differentiation was found by MANOVA between a number of lineages (Table 1). The high number of non-significant pair-wise comparisons in the MANOVA is likely to be caused by the limited sampling of certain clades in the phylogenetic tree [31].

Intermediate positions in morphospace found for certain lineages were highly indicative of the role of shape in evolution. Representatives of both Ablaberini + Sericini and species of Clade B are lacking in southern world continents (particularly in Australia), and the latter have invaded the Australian region (likely late during Tertiary) being present there only with a few species. Obviously, morphospace of Southern World Melolonthinae (i.e. Australian, as in Ahrens & Vogler [31] which included mainly Australian representatives) expanded due to the lack of these competitors. Wide overlap in morphospace with Clade B and Ablaberini + Sericini (Figures 2A–B, 3F–G) was the result. Accelerated rates of morphological divergence were observed with both data sets within this lineage. Early, fast divergence in body size of two major lineages preceded lower rates (Figure 6E), accompanied by medium to high rates of divergence in shape (Figure 6D). The increased rates of morphological divergence in Southern World Melolonthinae (Figure 6D–E) fit the scenario of rapid convergence in a framework of an ‘adaptive’ radiation in the Southern World, where occupation of ecological licenses may have been similar to Australian Marsupialia [82].

Rates of morphological divergence that were inferred from the densely sampled clade of Sericini might easily be influenced by a node-density effect [83], where lineages in less densely sampled clades appear to have lower rates of molecular evolution [84]. As phylogenetic independent contrasts are standardized over branch lengths, the rates of morphological divergence (Figure 6D–E) are also affected and should be considered with care.

### What shapes morphospace evolution?

Knowledge about the drivers of scarab shape divergence will greatly enhance our understanding of the evolutionary biology of this group of beetles. Whereas the analysis of the complete sampling allowed conclusions about general trends within Scarabaeidae, the investigation of subsets of the complete data revealed information about diverging traits between sister lineages. Although some measurements were likely to be correlated, as a whole they allowed differentiation between major lineages. Generally, conclusions that are drawn from the size-corrected data set are congruent with those from the uncorrected one, where size is contained. However, patterns of directed selection and rates of morphological divergence of uncorrected data showed a quite different and plausible signal from that of shape (Figure 6) indicating that body size itself has an important role in morphospace evolution.

Scarab shape morphospace (size-corrected data) was highly influenced by measurements of extremities and features linked with flight apparatus (EH; Figure 1). These traits are highly adaptive for burrowing and locomotion behavior and have undergone major changes in the evolution of pleurostict scarabs (Figure S2). Shorter and stouter forelegs in Dynastinae are suitable for burrowing in soil and organic matter; dorsoventral flattening of the body in Cetoniinae could be connected with the particular hovering flight behavior of the group (in particular among Cetoniini). Cetoniini beetles are able to target flowers in flight and land on them with high precision, an essential adaptation to anthophily. This ability is linked with key innovations of the elytral articulation and a lateral concave sinuation of the elytra for flight [85].

It is widely agreed that key innovations in phenotypic characters show evolutionary importance [86] and intensify diversification of a lineage [38], [87]. Metacoxal length (MCL) is conspicuously increased in Sericini, separating the mainly herbivorous lineage of Sericini from other herbivore scarabs (Figure S6). This lineage is significantly more speciose (ca. 4000 species) compared to its presumed sister lineage, the Ablaberini (ca. 200 species). Additionally, the Old World Sericini clade (ca. 3800 species) is more speciose compared to the Neotropical clade (ca. 200 species; here represented by *Astaena*). The influence of MCL on morphospace was conspicuous (Figure 3G, Figure S2H-I, Table S2 and S5), and the strong phylogenetic signal it exhibited, together with the decoupling of rates of morphological and molecular evolution, might possibly indicate an evolutionary shift and accompanying impact on morphospace evolution. Our results also showed that the K-value depends not only on the tree size [68], but also on sampling within the tree (Figure S3). It might, therefore, be questionable how useful it is to investigate the phylogenetic signal in order to infer directed selection on a certain trait. Nevertheless, even with only three sampled Sericini species, the K-value of MCL was the second highest value and in conjunction with the reconstruction of ancestral trait measures (Figure 5), it suggests strongly driven selection in relation to other traits towards a stabilization of an increased MCL within the lineage [68].

The link between high phylogenetic signal, and directed selection, possibly in combination with morphological key innovations, is not always evident. However, MCL was the only trait for which we found, based on evidence from a previous study [85], a trace of a physiologically linked counterpart. A subsequent maximum likelihood reconstruction of the size-corrected MCL (Figure 5A) on the tree revealed a strong shift of MCL's relative length (Figure 5A, left hand arrow) being linked with the secondary closure of the posterior opening of the metacoxal operculum, produced by the mesal metacoxal process and the posterior margin of the metacoxal plate ([85], Figure 5B–C, arrows). The presence of a mesal metacoxal process that originated among pleurostict chafers [85] allows a broader rotation of the hind limbs and a progressive enlargement of the MCL among the pleurosticts which could be explained with improved statics of the exoskeleton, in particular in context of the burrowing behavior. Evolutionary key innovations may have strong diverging influence on lineages in multivariate morphospace because they can promote evolutionary change in other traits [86]. The secondary closure of the posterior metacoxal opening produced by the extended mesal metacoxal process and the posterior margin of the metacoxal plate (Figure 5B–C, left side, [85]) is very likely an evolutionary key innovation [86]. This hypothesis is strongly supported by the subsequent increased rate of morphospace evolution (Figure 6C, arrow) and diversification. Its linkage with a substantial functional advantage enables Sericini to occupy new ecological space. Sericini species can burrow rapidly into sandy ground in case of danger by flapping their hind legs about 180° forwards and backwards (personal observation). The complete closure of the metacoxal ostium (Figure 5B–C; A, left hand arrow) enables the beetles to rotate the hind limb more anteriorly and, in combination with the increased metacoxal length, the locomotion statics of the body are improved for this burrowing behavior. A functional dependence of the metacoxal ostium and MCL is further supported as a reversal towards a slightly open metacoxal ostium, which occurred in the lineage of *Hymenoplia + Paratriodonta* ([85]; Figure 5A, right hand arrow). This is linked with a recurring slight reduction of MCL. Further functional consequences that increased locomotion statics might also be the observed reduction of sclerotization of the exoskeleton that presumably reduces body weight and possibly also physiological efficiency. Other morphological characters seem to support a hypothesis of this trend, such as the reduction of the elytral shelf in Sericini [85]. These hypotheses need further investigation and might be the subject of future research.

### The influence of size correction

Two different methods for removing isometric size from the data, the Burnaby Back Projection Method (BBPM) and linear regression against a size metric, only minor differed in morphospace patterns (Figures 2–3) and comparisons of relative shape divergence (Figure 5, Figure S5). The portion of total variation explained by the first two principal components was always slightly lower in the BBPM-data than in the data derived from linear regression. MANOVA on the BBPM-data recovered more significant pairwise lineage comparisons and mostly higher F-values (Tables 1–2, S10). However, significant results for the phylogenetic regression of feeding types in morphospace only resulted from the linear regression data (Table S13).

In the uncorrected data, divergent patterns of shape were less evident due to strong convergence of size and uneven distribution of variation (Table S8). In a few cases, size data improved the differentiation between groups or sister lineages (Tables 2–3). Size correction appears to be valuable for inference of patterns of shape variation [43]. As our study case has shown, it is informative to include size data, particularly when inferring rates of shape evolution, because patterns may be revealed that are in the same way relevant to niche formation and that may explain morphospace evolution from another perspective.

### Conclusion

Vast resources associated with angiosperm biomass seem to favor a hypothesis of reduced competition between adults. This is supported by the highly developed chemical communication of pleurosticts [22]–[25] that is used for aggregation, host location, and/or mate finding. But directed selection within the feeding types and strong rate shifts for some lineages indicated the opposite, at least for parts of the pleurostict tree. Significant shape divergence found between major lineages, combined with a lack of strong differentiation among younger and more closely related lineages such as the Sericini subgroups, indicated that the interpretation of results for pleurostict morphospace evolution, triggered by driven selection and competition, needs to be addressed at different time scales. The trend of convergence of feeding habits in multiple lineages indicate an evolutionary tendency that might be interpreted as resource partitioning which not in all cases is necessarily linked with morphospace divergence (e.g. Sericini). Striking morphospace divergence between some sister lineages with divergent feeding habits reveals that at least in the past (at the origin of these lineages) strong directed selection on morphospace was also likely to be linked with resource partitioning although being catalyzed by other factors such as feeding related locomotion behavior. But the same is true for scarabaeine dung beetles [17]. However, poor autecological knowledge of most pleurostict species and lacking community studies on assemblage level (competition acts only on individuals of all developmental stages in local assemblages) make it hard to investigate the linkage between divergence and competition in more detail. Therefore, further studies are needed to examine morphological divergence of pleurosticts and community composition at local scales to more rigorously investigate the question of competition. Conceivable hypothetical scenarios of competition might also include the issue of larval foraging and their competition for food and space, such as for dung beetles [15], because the larvae occupy an environment (soil) that is much more reduced in its dimensionality.

## Supporting Information

Figure S1 **Discrimination of phylogenetic sister clade lineages.** Barplots of the individual reassignment probabilities [%] from the discriminant analyses. Group membership priors are given under the plot by horizontal color bars. Rows refer to sister lineage subsets 1–5, columns show values for the uncorrected (left) and the size-corrected (BBPM; right) data sets.
(PDF)

Figure S2 **The drivers of morphospace divergence.** Biplots of PCA scores and loadings for the uncorrected and the size-corrected data sets: (A–C) Cetoniini + Valgini and Adoretini + Anomalini + Dynastinae, (D–F) Adoretini, Anomalini, and Dynastinae, (G–I) Clade B and Clade C, (J–L) Southern World Melolonthinae and Ablaberini + Sericini, and (M–O) Sericini subgroups. The groups are color-coded in the molecular phylogeny (Figure 3A). The percentage of variance explained by principal component 1 and 2 is given in the top right corner. Groups with more than 2 members are surrounded by a similarly colored hull. x-axis: PC1, y-axis: PC2. d =  mesh of the grid.
(PDF)

Figure S3 **Dependence of Bloomberg's et al.** [68] **descriptive K-statistic from the sampling.** Barplots of the K-values for all traits were calculated from the size-corrected data set for (A) the complete sampling (100 Sericini specimens) and reduced Sericini samplings with (B) 10 Sericini specimens and (C) 3 Sericini specimens. White bars indicate non-significance.
(PDF)

Figure S4 **Inference of branches in the trees were directed selection on the morphospace occurred.** The scatterplots illustrate morphological and molecular branch lengths for each branch in the trees. The ratios calculated from morphological to molecular branch lengths are quantified in the histograms above. Columns show values for internal branches and tips, rows show uncorrected and size-corrected data (BBPM). Dots of branches with significantly higher and lower morphological rates are indicated in red and blue, respectively.
(PDF)

Figure S5 **Main results with size corrected data from the linear regression method.** (A) Molecular phylogenetic tree [31], (B) tree with optimized branch lengths by the size-corrected data set from the linear regression method, (C) rates of morphological divergence (multivariate standardized phylogenetic independent contrasts) for the size-corrected data set mapped on the ultrametric phylogenetic tree showing relative divergence times, and (D) reconstruction of relative metacoxal length in ancestral nodes of the molecular phylogeny. The tips of the molecular tree (A) are color-coded for feeding habits (ANT  =  anthophilous, COP  =  coprophagous, HERB  =  herbivorous, SFU  =  sap/fluid utilizers, NF  =  not feeding, SAP  =  saprophagous). Branches in (B) with significantly lower (blue) and higher (red) morphological rates of evolution are colored respectively. Background shading indicates clade affiliation.
(PDF)

Table S1 **Full list of species in the study.** BMNH-shortcut, group affiliation, and assigned feeding habit.
(PDF)

Table S2 **PCA-loadings for PCs 1–3 of the analysis of the complete sampling.** BBPM-size-corrected (corr.) and uncorrected dataset (uncorr.). The last column shows K-values (phylogenetic signal) for every trait (size corrected data) for the complete sampling.
(PDF)

Table S3 **PCA-loadings for PCs 1–3 of the analysis of subset 1**. BBPM-size-corrected (corr.) and uncorrected dataset (uncorr.).
(PDF)

Table S4 **PCA-loadings for PCs 1–3 of the analysis of subset 2.** BBPM-size-corrected (corr.) and uncorrected dataset (uncorr.).
(PDF)

Table S5 **PCA-loadings for PCs 1–3 of the analysis of subset 3**. BBPM-size-corrected (corr.) and uncorrected dataset (uncorr.).
(PDF)

Table S6 **PCA-loadings for PCs 1–3 of the analysis of subset 4**. BBPM-size-corrected (corr.) and uncorrected dataset (uncorr.).
(PDF)

Table S7 **PCA-loadings for PCs 1–3 of the analysis of subset 5**. BBPM-size-corrected (corr.) and uncorrected dataset (uncorr.).
(PDF)

Table S8 **Percentage of total variation explained by principal components summing up to ≥95%.** BBPM-size-corrected and uncorrected dataset.
(PDF)

Table S9 **The impact of size.** Percentage of variation explained by size alone (PVESA) within the subsets.
(PDF)

Table S10 **Alternative size correction: F-values from non-parametric MANOVA of the complete sampling (excluding singletons) regarding 95% of total variation.** Values for the size-corrected dataset (with linear regression) are shown in the upper triangle, those for the uncorrected in the lower one. Significant differences (p<0.05) are highlighted in bold. Higher F-values for the same significant pairings are underlined in the respective triangle.
(PDF)

Table S11 **Alternative size correction: F-values from non-parametric MANOVA (Anderson 2001) of each subset (ss1–ss5, excluding singletons) regarding 95% of total variation.** Values for the size-corrected dataset (with linear regression) are shown in the upper triangle, those for the uncorrected in the lower one. Significant differences (p<0.05) are highlighted in bold.
(PDF)

Table S12 **Alternative size correction: Correlation between molecular and morphometric distance-matrices for specimens within one feeding type and the complete sampling (size-correction with linear regression).** Coefficients of determination and p-values from Mantel-tests.
(PDF)

Table S13 **Results of the phylogenetic least squares analyses.** Coefficients of determination and p-values are given for the uncorrected and both size-corrected data sets.
(PDF)

Script S1 **R-function for the calculation of multivariate standardized phylogenetic independent contrasts.**
(R) Click here for additional data file.
