# Supplementary material for: The Evolution of Morphospace in Phytophagous Scarab Chafers: No Competition - No Divergence?
Source: PLoS One. 2014 May 29;9(5):e98536. doi: 10.1371/journal.pone.0098536 (PMC4038600; doi:10.1371/journal.pone.0098536)
Supplement: Figure S1 — Discrimination of phylogenetic sister clade lineages. Barplots of the individual reassignment probabilities [%] from the discriminant analyses. Group membership priors are given under the plot by horizontal color bars. Rows refer to sister lineage subsets 1–5, columns show values for the uncorrected (left) and the size-corrected (BBPM; right) data sets. (PDF) [file pone.0098536.s001.pdf]

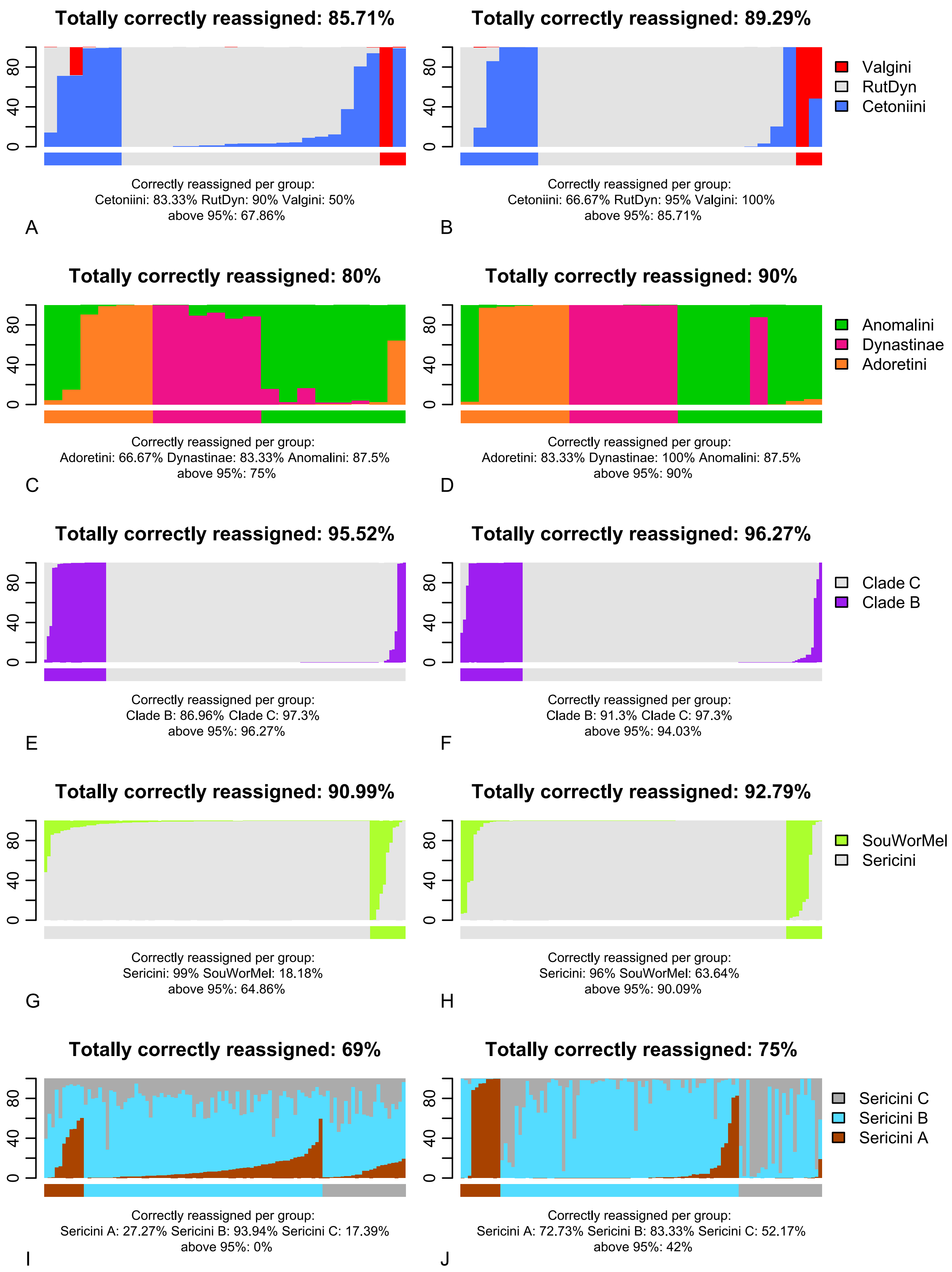
Totally correctly reassigned: 85.71%
Correctly reassigned per group:
Cetoniini: 83.33% RutDyn: 90% Valgini: 50%
above 95%: 67.86%
A
Totally correctly reassigned: 89.29%
Correctly reassigned per group:
Cetoniini: 66.67% RutDyn: 95% Valgini: 100%
above 95%: 85.71%
B
Valgini
RutDyn
Cetoniini
Totally correctly reassigned: 80%
Correctly reassigned per group:
Adoretini: 66.67% Dynastinae: 83.33% Anomalini: 87.5%
above 95%: 75%
C
Totally correctly reassigned: 90%
Correctly reassigned per group:
Adoretini: 83.33% Dynastinae: 100% Anomalini: 87.5%
above 95%: 90%
D
Anomalini
Dynastinae
Adoretini
Totally correctly reassigned: 95.52%
Correctly reassigned per group:
Clade B: 86.96% Clade C: 97.3%
above 95%: 96.27%
E
Totally correctly reassigned: 96.27%
Correctly reassigned per group:
Clade B: 91.3% Clade C: 97.3%
above 95%: 94.03%
F
Clade C
Clade B
Totally correctly reassigned: 90.99%
Correctly reassigned per group:
Sericini: 99% SouWorMel: 18.18%
above 95%: 64.86%
G
Totally correctly reassigned: 92.79%
Correctly reassigned per group:
Sericini: 96% SouWorMel: 63.64%
above 95%: 90.09%
H
SouWorMel
Sericini
Totally correctly reassigned: 69%
Correctly reassigned per group:
Sericini A: 27.27% Sericini B: 93.94% Sericini C: 17.39%
above 95%: 0%
I
Totally correctly reassigned: 75%
Correctly reassigned per group:
Sericini A: 72.73% Sericini B: 83.33% Sericini C: 52.17%
above 95%: 42%
J
Sericini C
Sericini B
Sericini A
