# Supplementary figures and images for: The Evolution of Morphospace in Phytophagous Scarab Chafers: No Competition - No Divergence?
Source: PLoS One. 2014 May 29;9(5):e98536. doi: 10.1371/journal.pone.0098536 (PMC4038600; doi:10.1371/journal.pone.0098536)

Uncorrected

Size Corrected with

Burnaby Back Projection Method

Linear Regression

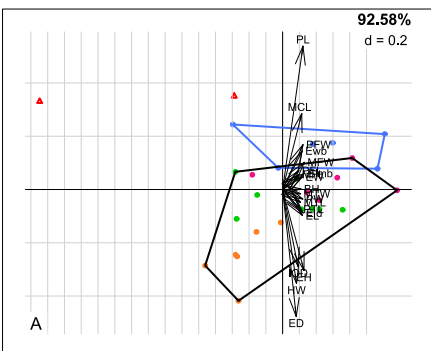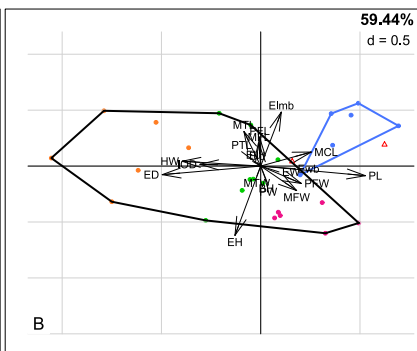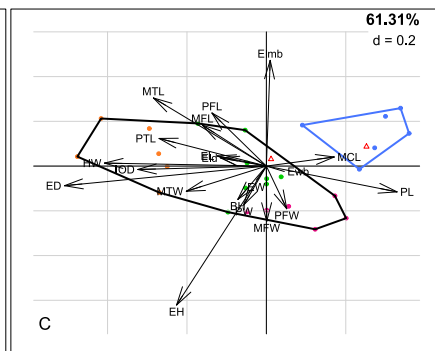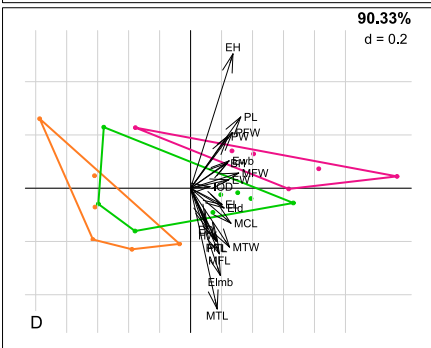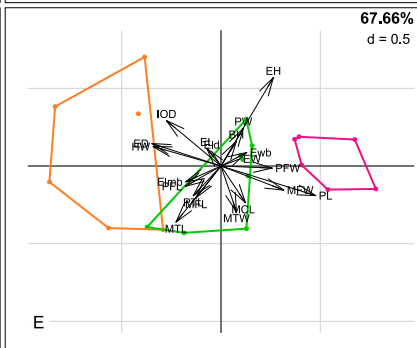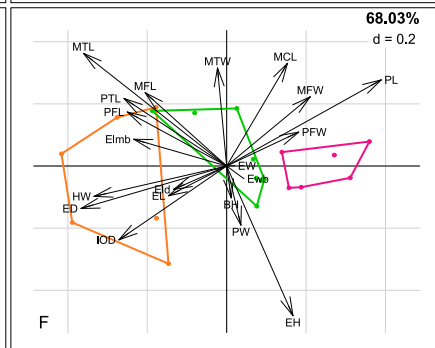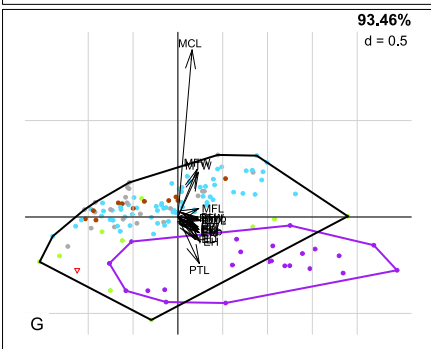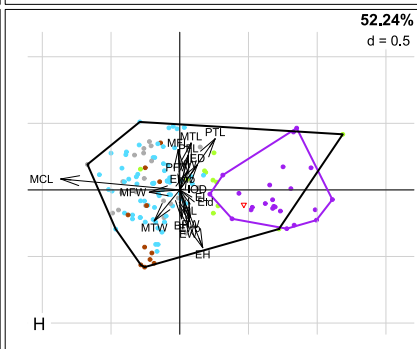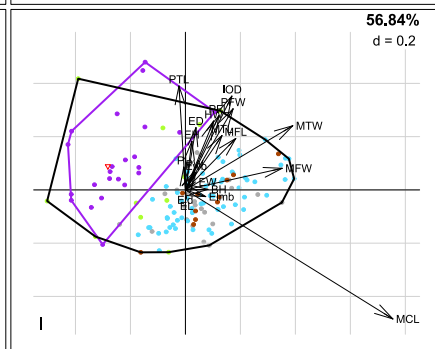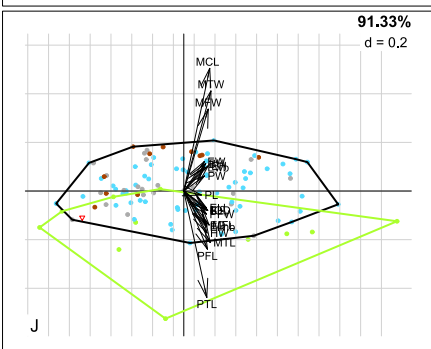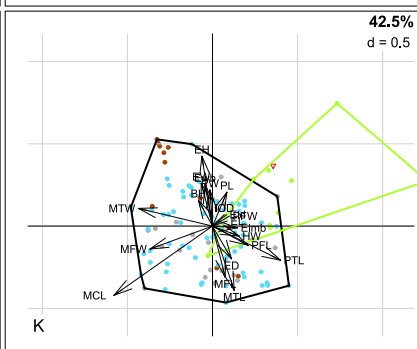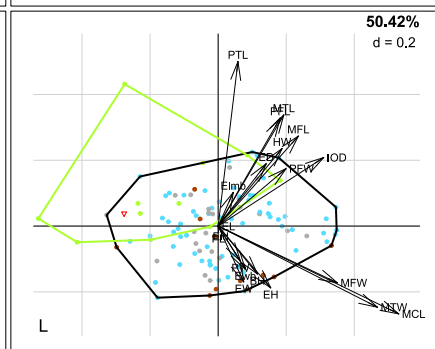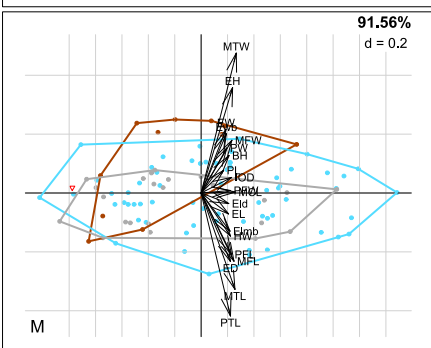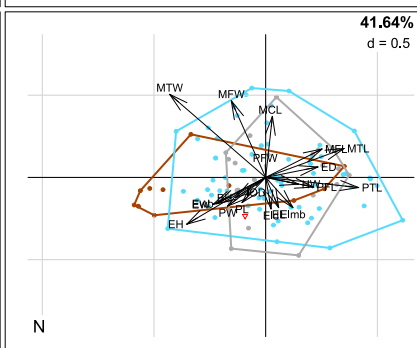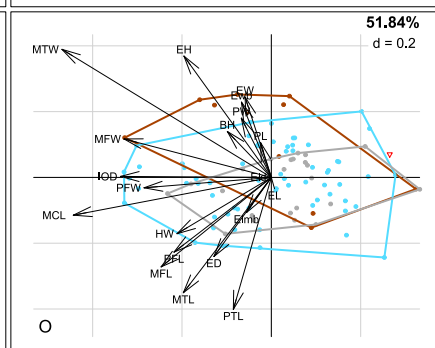

Supplement: Figure S2 — The drivers of morphospace divergence. Biplots of PCA scores and loadings for the uncorrected and the size-corrected data sets: (A–C) Cetoniini + Valgini and Adoretini + Anomalini + Dynastinae, (D–F) Adoretini, Anomalini, and Dynastinae, (G–I) Clade B and Clade C, (J–L) Southern World Melolonthinae and Ablaberini + Sericini, and (M–O) Sericini subgroups. The groups are color-coded in the molecular phylogeny (Figure 3A). The percentage of variance explained by principal component 1 and 2 is given in the top right corner. Groups with more than 2 members are surrounded by a similarly colored hull. x-axis: PC1, y-axis: PC2. d = mesh of the grid. (PDF) [file pone.0098536.s002.pdf]

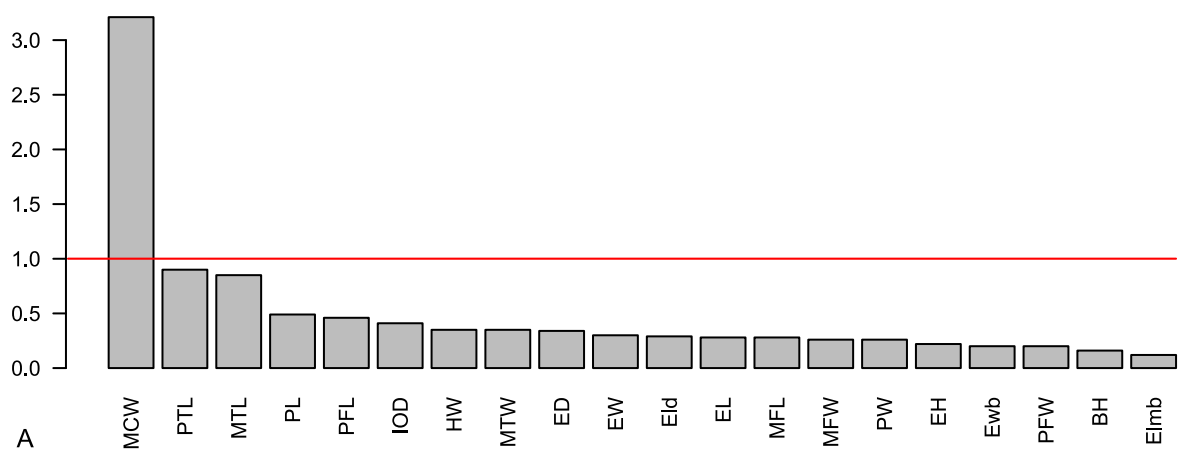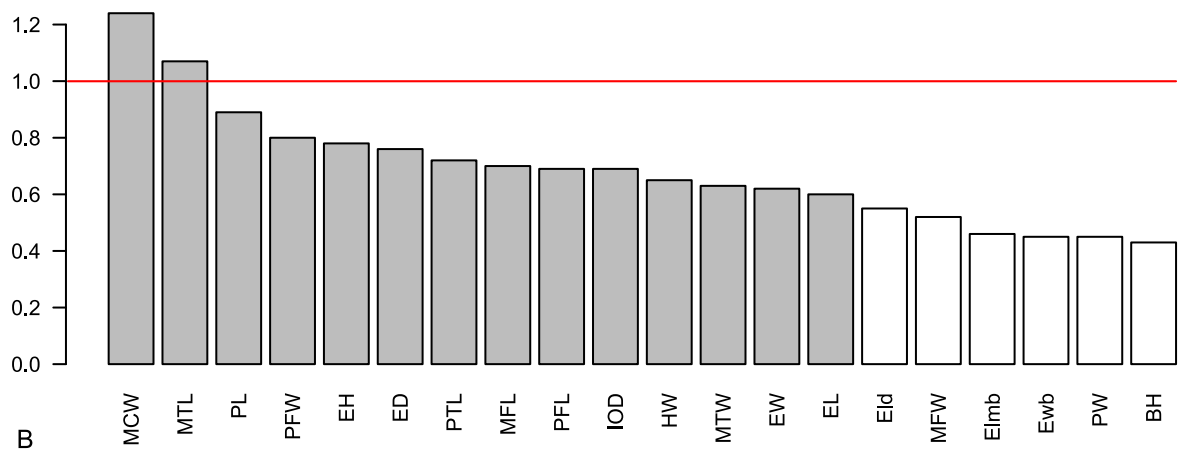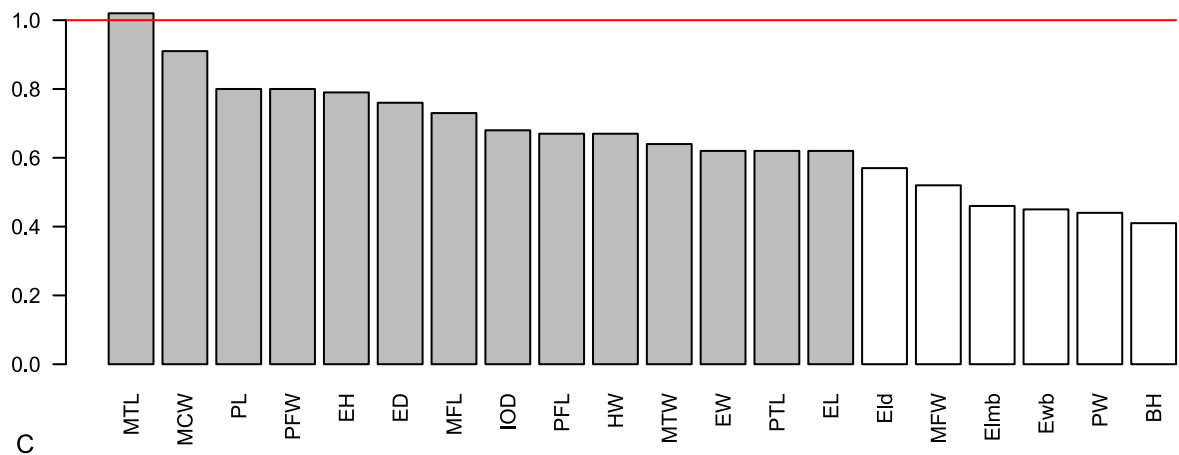

Supplement: Figure S3 — Dependence of Bloomberg's et al. [68] descriptive K-statistic from the sampling. Barplots of the K-values for all traits were calculated from the size-corrected data set for (A) the complete sampling (100 Sericini specimens) and reduced Sericini samplings with (B) 10 Sericini specimens and (C) 3 Sericini specimens. White bars indicate non-significance. (PDF) [file pone.0098536.s003.pdf]

uncorrected data

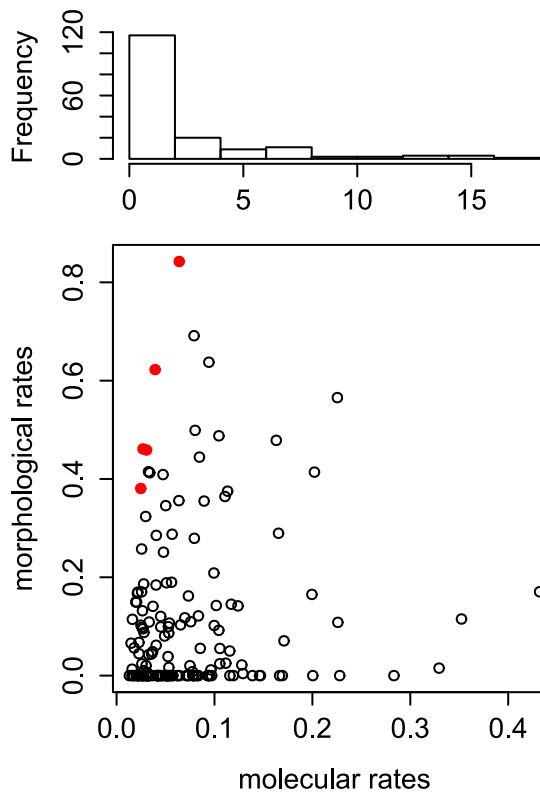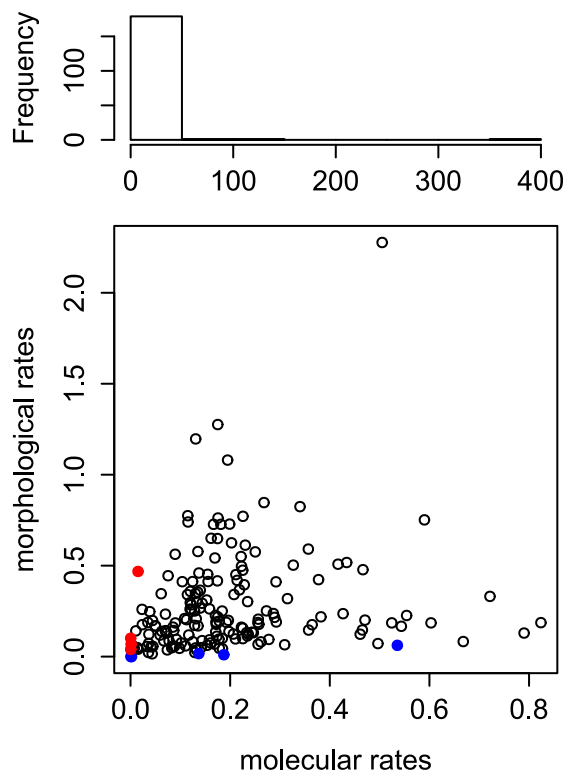

size-corrected data

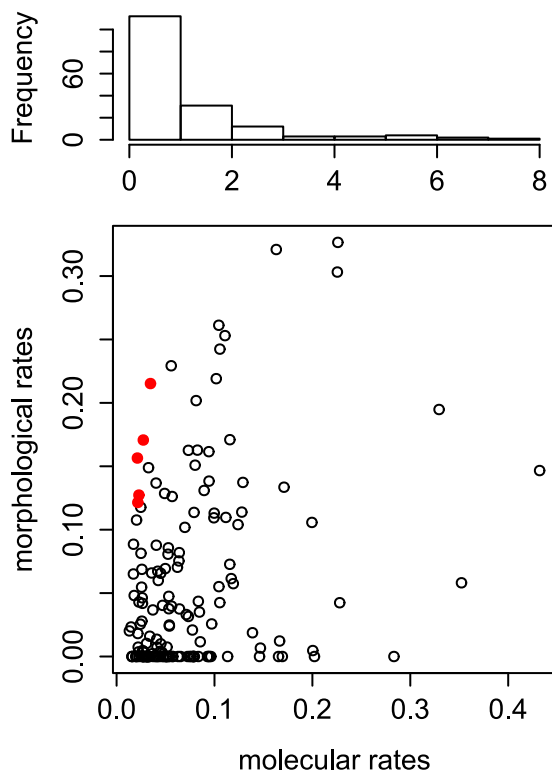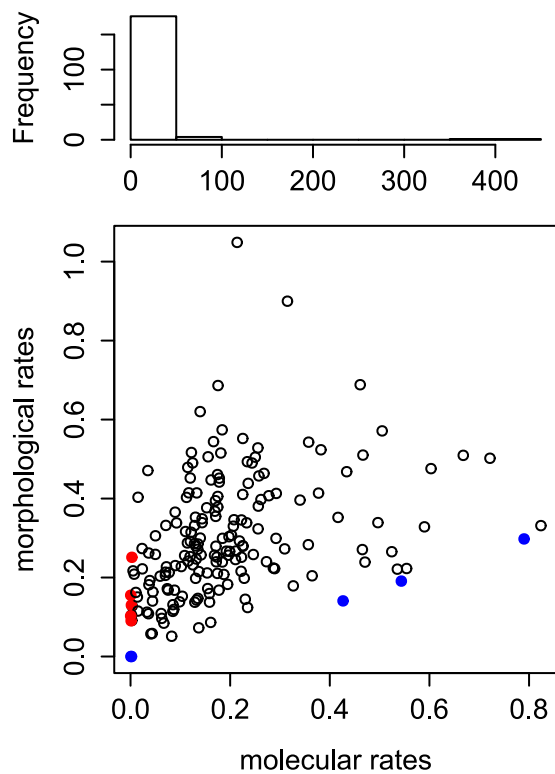

internal branches

tips

Supplement: Figure S4 — Inference of branches in the trees were directed selection on the morphospace occurred. The scatterplots illustrate morphological and molecular branch lengths for each branch in the trees. The ratios calculated from morphological to molecular branch lengths are quantified in the histograms above. Columns show values for internal branches and tips, rows show uncorrected and size-corrected data (BBPM). Dots of branches with significantly higher and lower morphological rates are indicated in red and blue, respectively. (PDF) [file pone.0098536.s004.pdf]

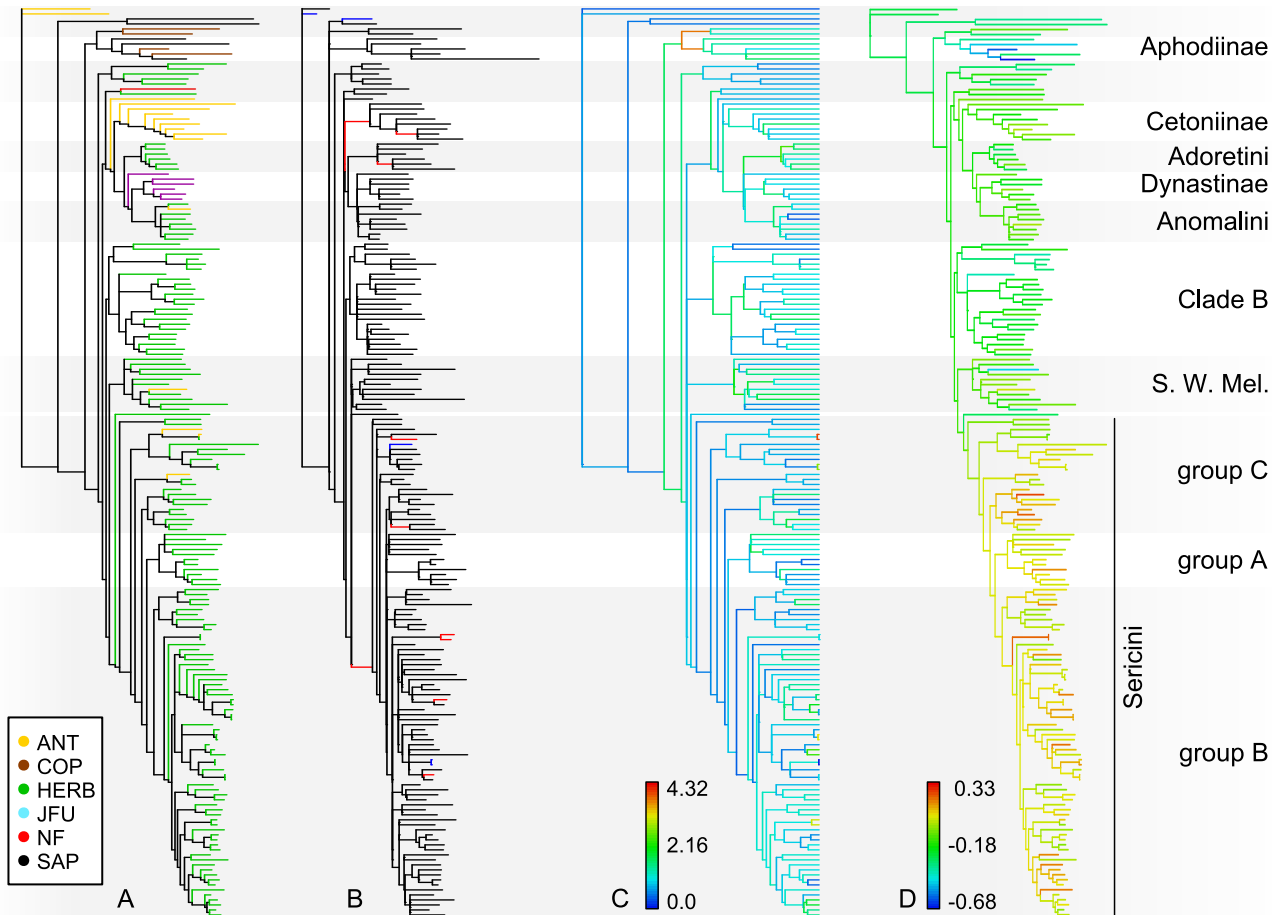

Supplement: Figure S5 — Main results with size corrected data from the linear regression method. (A) Molecular phylogenetic tree [31], (B) tree with optimized branch lengths by the size-corrected data set from the linear regression method, (C) rates of morphological divergence (multivariate standardized phylogenetic independent contrasts) for the size-corrected data set mapped on the ultrametric phylogenetic tree showing relative divergence times, and (D) reconstruction of relative metacoxal length in ancestral nodes of the molecular phylogeny. The tips of the molecular tree (A) are color-coded for feeding habits (ANT = anthophilous, COP = coprophagous, HERB = herbivorous, SFU = sap/fluid utilizers, NF = not feeding, SAP = saprophagous). Branches in (B) with significantly lower (blue) and higher (red) morphological rates of evolution are colored respectively. Background shading indicates clade affiliation. (PDF) [file pone.0098536.s005.pdf]
