# Supplementary material for: The Evolution of Morphospace in Phytophagous Scarab Chafers: No Competition - No Divergence?
Source: PLoS One. 2014 May 29;9(5):e98536. doi: 10.1371/journal.pone.0098536 (PMC4038600; doi:10.1371/journal.pone.0098536)
Supplement: Table S1 — Full list of species in the study. BMNH-shortcut, group affiliation, and assigned feeding habit. (PDF) [file pone.0098536.s006.pdf]

Table S1. Full list of species in the study. BMNH-shortcut, group affiliation, and assigned feeding habit.

Species	BMNH	clade affiliation (cf. Fig. 3A)	feeding habit (cf. Fig. 6A)
**Hybosoridae**			
Hybosorus illigeri (Reiche, 1853)	678457		SAP[11,7]
Phaeochrous sp.	678424		SAP
**Glaphyridae**			
Athypna carceli Castelnau, 1832	678442		ANT[3]
Eulasia (Rudeulasia) sp.	671327		ANT[3]
**Scarabaeidae**			
**Aegialiinae**			
Aegialia arenaria (F., 1787)	670855		SAP
**Chironinae**			
Chiron cyclindricus F., 1799	703555		SAP
**Aphodiinae**			
Dialytes sp.	703556		SAP
Oxyomus silvestris (Scopoli, 1772)	703657		COP
Aphodius (Trichaphodius) paradivisus Balthasar, 1960	703582		COP
**Scarabaeinae**			
Canthon viridis (Palisot de Beauvois, 1885)	679736		COP
Sisyphus fasciculatus Boheman, 1857	679906		COP
**Cetoniinae**			
**Cetoniini**			
Cetonia aurata aurata (L., 1761)	678451	Cetoniini	ANT[8]
Chiloloba acuta (Wiedemann, 1823)	677849	Cetoniini	ANT[2]
Heteorrhina micans (Guerin, 1840)	677846	Cetoniini	ANT
Heterocnemis graeca (Brullé)	678459	Cetoniini	ANT
Netocia cuprea (F., 1775)	678460	Cetoniini	ANT
Oxythyrea cinctella (Schaum, 1841)	678461	Cetoniini	ANT[10]
**Valgini**			
Microvalgus sp.	671321	Valgini	ANT
Valgus hemipterus (L., 1758)	678441	Valgini	ANT[8]
**Dynastinae**			
Alissonotum simile Arrow, 1910	670853	Dynastinae / Clade A	SFU
Cheiroplatys latipes (Guérin, 1830)	670926	Dynastinae / Clade A	SFU
Heteronychus lioderes Redtenbacher, 1867	677863	Dynastinae / Clade A	SFU[8]
Pentodon idiota (Herbst, 1789)	678452	Dynastinae / Clade A	SFU
Phyllognathus dionysius (Fabricius, 1792)	670907	Dynastinae / Clade A	SFU
Pimelopus dubius dubius Blackburn, 1885	670927	Dynastinae / Clade A	SFU
**Melolonthinae**			
**Melolonthini**			
Lepidiota albistigma Burmeister, 1855	670909	Clade B	HERB
Lepidiota stradbrokensis Lea, 1919	671319	Clade B	HERB
Melolontha melolontha (L., 1758)	670877	Clade B	HERB[12]
**Rhizotrogini**			
Amphimallon solstitiale (Linnaeus, 1758)	678446	Clade B	HERB
Aplidia transversa peloponnisica Petrovitz, 1971	678410	Clade B	HERB
Empecta sicardi Dewailly, 1950	671479	Clade B	HERB
Holotrichia seticollis Moser, 1912	677874	Clade B	HERB
Hoplochelus piliger (Blanchard, 1850)	671422	Clade B	HERB

Idionycha excisa Arrow, 1932	677857	Clade B	HERB
Sophrops sp.	677844	Clade B	HERB
**Schizonychini**			
Schizonycha fuscescens Blanchard, 1850	677896	Clade B	HERB[8]
**Diplotaxini**			
Apogonia sp.	671353	Clade B	HERB[8]
Apogonia sp.	671463	Clade B	HERB[8]
Ceratogonia bicornuta (Kolbe, 1899)	671439	Clade B	HERB
Dichecephala ovata (Fahraeus, 1857)	671461	Clade B	HERB
**Automoliini**			
Automolius humilis (Blanchard, 1850)	671320	Southern World Mel.	HERB
**Pachydemini**			
Buettikeria echinocephala Nikolajev, 2003	747069	Clade B	HERB
**Chasmatopterini**			
Chasmatopterus sp.	694788	Clade B	HERB
Colpochila sp.	670928	Southern World Mel.	HERB
**Enariini**			
Apiencya sp.	671495	Clade B	HERB
Cherbezatina strigosa Lacroix, 1993	671414	Clade B	HERB
Enaria boissayei Dewailly, 1950	671494	Clade B	HERB
Euthora sp.	671497	Clade B	HERB
Eutrichesis pilosicollis Waterhouse, 1882	671423	Clade B	HERB
Varencya sp.	671503	Clade B	HERB
**Heteronycini**			
Heteronyx sp.	670839	Southern World Mel.	HERB[13]
Heteronyx sp.	671323	Southern World Mel.	HERB[13]
Melolonthine gen. sp.	671352	Southern World Mel.	HERB
**Scitaliini**			
Homalotropus sp.	670840	Southern World Mel.	HERB
Scitala aureorufa (Blanchard, 1850)	670846	Southern World Mel.	HERB
**Macrodactylini**			
Isonychus sp.	670885		ANT[4]
**Liparetrini**			
Liparetrus sp.	671322	Southern World Mel.	HERB
Sericoides cf. sp.	671333	Southern World Mel.	HERB
**Maechidiini**			
Maechidius sp.	670841	Southern World Mel.	HERB
**Phyllotocini**			
Phyllotocus sp.	703645	Southern World Mel.	ANT[5]
**Pachypodini**			
Pachypus candidae (Petagna, 1786)	694786		NF
**Sericini group A**			
Ablaberoides sp.	671485	Sericini group A	HERB[1]
Allokotarsa clypeata Peringuey, 1904	671402	Sericini group A	HERB[1]
Idaeserica sp.	671445	Sericini group A	HERB[1]
Trochalina gen. sp.	671448	Sericini group A	HERB[1]
Trochaloserica sp.	671486	Sericini group A	HERB[1]
Trochalus sp.	670862	Sericini group A	HERB[1]
Trochalus sp.	670865	Sericini group A	HERB[1]
Trochalus sp.	671416	Sericini group A	HERB[1]
Trochalus sp.	671429	Sericini group A	HERB[1]
Trochalus sp.	671433	Sericini group A	HERB[1]
Trochalus sp.	671453	Sericini group A	HERB[1]
**Sericini group B**			
Anomalophylla sp.	678368	Sericini group B	HERB[1]

Anomalophylla tristicula Reitter, 1887	678371	Sericini group B	HERB[1]
Chrysoserica stebnickae Ahrens, 2002	670890	Sericini group B	HERB[1]
Euserica villarreali Baraud, 1975	747066	Sericini group B	HERB[1]
Gynaecoserica variipennis variipennis (Moser, 1916)	678396	Sericini group B	HERB[1]
Lamproserica sp.	670867	Sericini group B	HERB[1]
Lamproserica sp.	671446	Sericini group B	HERB[1]
Lamproserica sp.	671484	Sericini group B	HERB[1]
Lepiserica sp.	671452	Sericini group B	HERB[1]
Maladera affinis (Blanchard, 1850)	670849	Sericini group B	HERB[x]
M. basalis (Moser, 1915)	671358	Sericini group B	HERB[1]
M. burmeisteri (Brenske, 1898)	671354	Sericini group B	HERB[1]
M. cardoni (Brenske, 1896)	677841	Sericini group B	HERB[1]
M. cf *fuscescens* (Moser, 1917)	671454	Sericini group B	HERB[1]
M. fuscescens (Moser, 1917)	671450	Sericini group B	HERB[1]
M. himalayica incola Ahrens, 2004	678407	Sericini group B	HERB[x]
M. holosericea (Scopoli, 1772)	670868	Sericini group B	HERB[1]
M. iridescens (Blanchard, 1850)	671366	Sericini group B	HERB[1]
M. joachimi Ahrens, 2004	671329	Sericini group B	HERB[x]
M. kanarana (Moser, 1918)	671361	Sericini group B	HERB[1]
M. quinquidens (Brenske, 1896)	678417	Sericini group B	HERB[x]
M. significans (Brenske, 1898)	671357	Sericini group B	HERB[1]
M. simlana (Brenske, 1898)	678404	Sericini group B	HERB[x]
M. simlana (Brenske, 1898)	670891	Sericini group B	HERB[x]
M. thomsoni (Brenske, 1894)	678412	Sericini group B	HERB[x]
Maladera sp.	671328	Sericini group B	HERB[1]
Maladera sp.	671344	Sericini group B	HERB[1]
Maladera sp.	671356	Sericini group B	HERB[1]
Maladera sp.	671360	Sericini group B	HERB[1]
Maladera sp.	671364	Sericini group B	HERB[1]
Maladera sp.	671449	Sericini group B	HERB[1]
Maladera sp.	671488	Sericini group B	HERB[1]
Microserica gandakiensis Ahrens, 1998	678403	Sericini group B	HERB[1]
M. interrogator (Arrow, 1946)	678398	Sericini group B	HERB[1]
M. pruinosa (Hope, 1831)	670898	Sericini group B	HERB[x]
M. soppongensis Ahrens, 2005	670858	Sericini group B	HERB[1]
Microserica cf. sp.	703024	Sericini group B	HERB[1]
Neoserica sp.	671337	Sericini group B	HERB[1]
Neoserica sp.	671343	Sericini group B	HERB[1]
Neoserica sp.	671345	Sericini group B	HERB[1]
Neoserica sp.	671346	Sericini group B	HERB[1]
Neoserica sp.	671347	Sericini group B	HERB[1]
Neoserica sp.	671348	Sericini group B	HERB[1]
Neoserica sp.	671349	Sericini group B	HERB[1]
Neoserica sp.	671351	Sericini group B	HERB[1]
Neoserica sp.	671340	Sericini group B	HERB[1]
Neoserica sp. 246	703021	Sericini group B	HERB[1]
Nepaloserica mustangia Ahrens & Sabatinelli, 1996	670894	Sericini group B	HERB[1]
N. schmidti Ahrens & Sabatinelli, 1996	678402	Sericini group B	HERB[x]
Nepaloserica sp.	670901	Sericini group B	HERB[1]
Nepaloserica sp.	670896	Sericini group B	HERB[1]
Neuroserica fulvescens (Blanchard, 1850)	671426	Sericini group B	HERB[1]
Neuroserica mashona Peringuey, 1904	671442	Sericini group B	HERB[1]
Nipponoserica koltzei (Reitter, 1897)	678372	Sericini group B	HERB[1]
Oxyserica pygidialis pygidialis (Brenske, 1900)	670893	Sericini group B	HERB[x]
Pachyserica olafi Ahrens, 2004	678395	Sericini group B	HERB[x]
Serica benesi Ahrens, 2005	678375	Sericini group B	HERB[1]

S. brunnea (L., 1758)	670861	Sericini group B	HERB[1]
S. heydeni Reitter, 1896	678366	Sericini group B	HERB[1]
S. intermixta Blatchley. 1910	678394	Sericini group B	HERB[1]
S. loxia Dawson, 1920	678391	Sericini group B	HERB[1]
S. mystaca Dawson, 1920	678384	Sericini group B	HERB[1]
S. tukucheana Ahrens, 1999	670899	Sericini group B	HERB[x]
Serica sp.	678376	Sericini group B	HERB[1]
Serica sp.	671336	Sericini group B	HERB[1]
Tetraserica sp.	671350	Sericini group B	HERB[1]
**Sericini group C**			
Astaena sp.	671334	Sericini group C	HERB[1]
Astaena sp.	671341	Sericini group C	HERB[1]
Comaserica crinita (Burmeister, 1855)	671413	Sericini group C	HERB[1]
Comaserica sp.	671397	Sericini group C	HERB[1]
Comaserica sp.	671470	Sericini group C	HERB[1]
Comaserica sp.	671401	Sericini group C	HERB[1]
Hellaserica elongata (Reitter, 1887)	678381	Sericini group C	HERB[x]
Hymenoplia fulvipennis Blanchard, 1850	747061	Sericini group C	ANT[x]
H. lineolata Blanchard, 1850	747067	Sericini group C	ANT[x]
Hyposerica sp.	671398	Sericini group C	HERB[1]
Hyposerica sp.	671474	Sericini group C	HERB[1]
Hyposerica sp.	671469	Sericini group C	HERB[1]
Hyposerica sp.	671410	Sericini group C	HERB[1]
Hyposerica sp.	678454	Sericini group C	HERB[1]
Omaloplia nigromarginata (Herbst, 1785)	747063	Sericini group C	HERB[1]
O. ruricola (F., 1775)	747065	Sericini group C	HERB[1]
Paratriodonta romana (Brenske, 1890)	670857	Sericini group C	ANT
Triodontella sp.	671408	Sericini group C	HERB[1]
Triodontella sp.	671415	Sericini group C	HERB[1]
Triodontella sp.	671440	Sericini group C	HERB[1]
Triodontella sp.	671458	Sericini group C	HERB[1]
Triodontella sp.	671483	Sericini group C	HERB[1]
Triodontella sp.	671489	Sericini group C	HERB[1]
**Ablaberini**			
Cyrtocamenta sp.	671406	Ablaberini	HERB
**Diphycerini**			
Diphycerus sp.	677856		HERB
**Hopliinae**			HERB
Apomorphochelus sp.	671482		HERB
Echyra umbrina (Fairmaire, 1886)	671471		HERB
Madohopliini gen. sp.	671507		HERB
Michaeloplia montana Lacroix, 1998	671481		HERB
Paramorphochelus agricola (Lebis, 1961)	671505		HERB
**Rutelinae**			
**Adoretini**			
Adoretus sp.	677872	Adoretini / Clade A	HERB[8]
Adoretus sp.	677883	Adoretini / Clade A	HERB[8]
Adoretus versutus Harold, 1869	677880	Adoretini / Clade A	HERB[8]
Lepadoretus ovatoides Machatschke, 1961	671466	Adoretini / Clade A	HERB
Prodoretus truncatus Arrow, 1915	671508	Adoretini / Clade A	HERB
Trigonostomum mucoreum Burmeister, 1844	671418	Adoretini / Clade A	HERB
**Anomalini**			
Anisoplia dispar Erichson, 1847	678448	Anomalini / Clade A	ANT[6]

Anomala biharensis Arrow, 1917	677861	Anomalini / Clade A	HERB[8]
A. bilobata Arrow, 1912	677897	Anomalini / Clade A	HERB[8]
A. cantori (Hope, 1839)	677825	Anomalini / Clade A	HERB[8]
A. dorsalis (F., 1775)	677902	Anomalini / Clade A	HERB[8]
Blithopertha lineolata (Fischer, 1823)	671502	Anomalini / Clade A	HERB
Mimela siliguria Arrow, 1917	677833	Anomalini / Clade A	HERB
Phyllopertha horticola (L., 1758)	678428	Anomalini / Clade A	HERB[9]

Feeding type references: [x] = personal observation.

1. Ahrens, D. (2005) The phylogeny of Sericini and their position within the Scarabaeidae based on morphological characters (Coleoptera: Scarabaeidae). *Systematic Entomology* 31, 113-144.

2. Arrow G.J. (1910) *The Fauna of British India (Including Ceylon and Burma) Lamellicornia I.*Taylor & Francis. London, 1-322.

3. Dafni, A., Bernhardt, P., Shmida, A., Ivri, B. Y., Greenbaum, S., O'Toole, C., & Losito, L. (1990) Red bowl-shaped flowers: convergence for beetle pollination in the Mediterranean region. *Israel Journal of Botany*, 39, 81–92.

4. Gomes, R., & Pinheiro, M. C. B. (2007) Biologia floral de *Pouteria venosa* (Matius) Baehni (Sapotaceae) na restinga de Maricá–RJ. *Revista Brasileira de Biociências*, 5(supl. 1), 108–110.

5. Hingston, A. B., & Potts, B. M. (1998) Floral visitors of *Eucalyptus globulus* subsp. *globulus* in eastern Tasmania. *Tasforets*, 10, 125–139.

6. Micó, E., Sanmartín, I., & Galante, E. (2009) Mediterranean diversification of the grass-feeding Anisopliina beetles (Scarabaeidae, Rutelinae, Anomalini) as inferred by bootstrap-averaged dispersal-vicariance analysis. *Journal of Biogeography*, 36(3), 546–560.

7. Ocampo, F. C. (2002) Hybosorids of the United States and Expanding Distribution of the Introduced Species *Hybosorus illigeri* (Coleoptera: Scarabaeoidea: Hybosoridae). *Annals of the Entomological Society of America*, 95(3), 316–322.

8. Ritcher, P. O. (1958) Biology of Scarabaeidae. *Annual review of Entomology* 3, 311–333.

9. Rittershaus, K. (1927) Studien zur Morphologie und Biologie von *Phyllopertha horticola* L. und *Anomala aenea* Geer. (Coleopt.). *Zeitschrift für Morphologie und Ökologie der Tiere*, 18–408.

10. Rittner, O., & Sabatinelli, G. (2010) The genus *Oxythyrea* Mulsant in Israel (Coleoptera: Scarabaeidae: Cetoniinae: Cetoniini). *Israel Journal of Entomology*, 40, 11–19.

11. Rozas, L., Avila, J. M., & Sánchez-Piñero, F. (1991) Observación de hábitos depredadores en *Hybosorus illigeri* Reiche, 1853 (Coleóptera, Scarabaeoidea, Hybosoridae). *Boletín de la Asociación española de Entomología*, 15, 111-115.

12. Scholtz, C.H., Grebennikov, V.V., (2005) Scarabaeoidea Latreille, 1802. In: Beutel, R.G., Leschen, R.A.B. (Eds.), *Coleoptera, Beetles. vol. 1: Morphology and Systematics (Archostemata, Adephaga, Myxophaga, Polyphaga partim). Handbook of Zoology. vol. IV Arthropoda: Insecta part 38*. W. de Gruyter-Berlin, New York, pp. 367–426.

13. Ward, A. L., & Rogers, D. J. (2006) Population ecology of *Heteronyx piceus* (Coleoptera: Scarabaeidae) in a peanut/maize cropping system. *Bulletin of entomological research* 96(02), 129-136.
