# Supplementary material for: The Evolution of Morphospace in Phytophagous Scarab Chafers: No Competition - No Divergence?
Source: PLoS One. 2014 May 29;9(5):e98536. doi: 10.1371/journal.pone.0098536 (PMC4038600; doi:10.1371/journal.pone.0098536)
Supplement: Table S2 — PCA-loadings for PCs 1–3 of the analysis of the complete sampling. BBPM-size-corrected (corr.) and uncorrected dataset (uncorr.). The last column shows K-values (phylogenetic signal) for every trait (size corrected data) for the complete sampling. (PDF) [file pone.0098536.s007.pdf]

**Table S2. PCA-loadings for PCs 1-3 of the analysis of the complete sampling.** BBPM-size-corrected (corr.) and uncorrected dataset (uncorr.). The last column shows K-values (phylogenetic signal) for every trait (size corrected data) for the complete sampling.

| **uncorr.** | **PC1** | **PC2** | **PC3** | **corr.** | **PC1** | **PC2** | **PC3** | **K-statistic** |
|---|---|---|---|---|---|---|---|---|
| *EL* | 0.23 | 0.01 | -0.19 | *EL* | -0.02 | 0.17 | 0.23 | 0.28 |
| *PL* | 0.21 | 0.15 | 0.01 | *PL* | -0.14 | 0.02 | -0.36 | 0.49 |
| *Eld* | 0.23 | 0.03 | -0.21 | *Eld* | -0.04 | 0.19 | 0.19 | 0.29 |
| *Elmb* | 0.23 | -0.02 | -0.02 | *Elmb* | 0.01 | 0.01 | 0.26 | 0.12 |
| *EW* | 0.22 | 0 | -0.2 | *EW* | 0 | 0.19 | 0 | 0.3 |
| *Ewb* | 0.23 | 0.05 | -0.11 | *Ewb* | -0.06 | 0.1 | -0.09 | 0.2 |
| *PW* | 0.22 | 0.07 | -0.18 | *PW* | -0.07 | 0.18 | -0.07 | 0.26 |
| *BH* | 0.22 | -0.03 | -0.21 | *BH* | 0.02 | 0.2 | 0.01 | 0.16 |
| *EH* | 0.23 | 0.08 | -0.57 | *EH* | -0.08 | 0.55 | -0.02 | 0.22 |
| *HW* | 0.21 | 0.06 | 0.01 | *HW* | -0.06 | 0.01 | 0.18 | 0.35 |
| *IOD* | 0.21 | 0.11 | -0.13 | *IOD* | -0.1 | 0.16 | -0.06 | 0.41 |
| *ED* | 0.22 | 0.05 | 0.06 | *ED* | -0.06 | -0.06 | 0.34 | 0.34 |
| *PTL* | 0.24 | 0.27 | 0.33 | *PTL* | -0.29 | -0.33 | 0.03 | 0.9 |
| *PFL* | 0.23 | 0.09 | 0.27 | *PFL* | -0.1 | -0.26 | 0.09 | 0.46 |
| *PFW* | 0.22 | 0.07 | 0.11 | *PFW* | -0.07 | -0.09 | -0.34 | 0.2 |
| *MTL* | 0.26 | 0.08 | 0.37 | *MTL* | -0.11 | -0.4 | 0.21 | 0.85 |
| *MTW* | 0.24 | -0.14 | 0.13 | *MTW* | 0.12 | -0.16 | -0.48 | 0.35 |
| *MFL* | 0.23 | 0 | 0.3 | *MFL* | -0.01 | -0.31 | 0.12 | 0.28 |
| *MFW* | 0.23 | -0.19 | 0.1 | *MFW* | 0.17 | -0.12 | -0.35 | 0.26 |
| *MCW* | 0.19 | -0.89 | 0.04 | *MCW* | 0.89 | -0.05 | 0.12 | 3.21 |
