# Supplementary material for: The Evolution of Morphospace in Phytophagous Scarab Chafers: No Competition - No Divergence?
Source: PLoS One. 2014 May 29;9(5):e98536. doi: 10.1371/journal.pone.0098536 (PMC4038600; doi:10.1371/journal.pone.0098536)
Supplement: Table S3 — PCA-loadings for PCs 1–3 of the analysis of subset 1. BBPM-size-corrected (corr.) and uncorrected dataset (uncorr.). (PDF) [file pone.0098536.s008.pdf]

Table S3. PCA-loadings for PCs 1-3 of the analysis of subset 1. BBPM-size-corrected (corr.) and uncorrected dataset (uncorr.).

**uncorr.**	**PC1**	**PC2**	**PC3**	**corr.**	**PC1**	**PC2**	**PC3**
EL	0.24	0.1	-0.1	*EL*	0.04	0.04	0.28
PL	0.24	-0.54	0.2	*PL*	-0.53	-0.09	-0.15
Eld	0.25	0.09	-0.09	*Eld*	0.02	0.03	0.28
Elmb	0.26	-0.06	-0.56	*Elmb*	-0.1	0.48	0.53
EW	0.24	-0.05	0.04	*EW*	-0.09	-0.05	0.13
Ewb	0.23	-0.15	0.03	*Ewb*	-0.17	-0.02	0.16
PW	0.24	0.04	0.14	*PW*	-0.04	-0.17	0.22
BH	0.22	0	0.14	*BH*	-0.03	-0.14	0.08
EH	0.25	0.3	0.53	*EH*	0.13	-0.62	0.21
HW	0.17	0.35	-0.08	*HW*	0.4	0.04	-0.09
IOD	0.18	0.29	-0.05	*IOD*	0.31	0.02	0.03
ED	0.16	0.48	0.02	*ED*	0.5	-0.08	-0.07
PTL	0.21	0.07	-0.13	*PTL*	0.1	0.13	-0.21
PFL	0.19	-0.07	-0.22	*PFL*	0.01	0.25	-0.16
PFW	0.24	-0.17	0.19	*PFW*	-0.2	-0.16	-0.15
MTL	0.21	0.05	-0.32	*MTL*	0.08	0.31	-0.2
MTW	0.21	0.02	0.12	*MTW*	0.02	-0.09	-0.4
MFL	0.19	-0.06	-0.16	*MFL*	0.01	0.2	-0.2
MFW	0.26	-0.1	0.22	*MFW*	-0.18	-0.22	-0.12
MCW	0.23	-0.29	-0.06	*MCW*	-0.25	0.13	-0.16
