# Supplementary material for: The Evolution of Morphospace in Phytophagous Scarab Chafers: No Competition - No Divergence?
Source: PLoS One. 2014 May 29;9(5):e98536. doi: 10.1371/journal.pone.0098536 (PMC4038600; doi:10.1371/journal.pone.0098536)
Supplement: Table S4 — PCA-loadings for PCs 1–3 of the analysis of subset 2. BBPM-size-corrected (corr.) and uncorrected dataset (uncorr.). (PDF) [file pone.0098536.s009.pdf]

**Table S4. PCA-loadings for PCs 1-3 of the analysis of subset 2.** BBPM-size-corrected (corr.) and uncorrected dataset (uncorr.).

| **uncorr.** | **PC1** | **PC2** | **PC3** | **corr.** | **PC1** | **PC2** | **PC3** |
|---|---|---|---|---|---|---|---|
| *EL* | 0.2 | 0.06 | -0.23 | *EL* | 0.07 | 0.11 | 0.17 |
| *PL* | 0.32 | -0.27 | 0.5 | *PL* | -0.48 | -0.19 | -0.23 |
| *Eld* | 0.22 | 0.07 | -0.23 | *Eld* | 0.05 | 0.09 | 0.12 |
| *Elmb* | 0.19 | 0.33 | -0.21 | *Elmb* | 0.18 | -0.1 | 0.29 |
| *EW* | 0.25 | -0.03 | -0.06 | *EW* | -0.1 | 0.05 | 0.04 |
| *Ewb* | 0.25 | -0.1 | -0.04 | *Ewb* | -0.13 | 0.09 | 0.02 |
| *PW* | 0.24 | -0.19 | -0.17 | *PW* | -0.11 | 0.24 | 0.03 |
| *BH* | 0.24 | -0.09 | -0.16 | *BH* | -0.08 | 0.16 | 0.14 |
| *EH* | 0.27 | -0.5 | -0.34 | *EH* | -0.26 | 0.57 | 0.07 |
| *HW* | 0.11 | 0.15 | -0.26 | *HW* | 0.34 | 0.13 | -0.07 |
| *IOD* | 0.13 | -0.01 | -0.32 | *IOD* | 0.27 | 0.29 | -0.41 |
| *ED* | 0.1 | 0.13 | -0.27 | *ED* | 0.35 | 0.14 | 0.28 |
| *PTL* | 0.17 | 0.2 | 0.13 | *PTL* | 0.14 | -0.19 | -0.29 |
| *PFL* | 0.16 | 0.2 | 0.05 | *PFL* | 0.18 | -0.13 | -0.33 |
| *PFW* | 0.27 | -0.21 | 0.23 | *PFW* | -0.26 | -0.01 | -0.33 |
| *MTL* | 0.17 | 0.45 | 0.08 | *MTL* | 0.23 | -0.36 | -0.02 |
| *MTW* | 0.25 | 0.22 | 0.14 | *MTW* | -0.08 | -0.29 | 0.47 |
| *MFL* | 0.19 | 0.25 | 0.07 | *MFL* | 0.12 | -0.2 | -0.1 |
| *MFW* | 0.31 | -0.06 | 0.21 | *MFW* | -0.32 | -0.16 | 0.09 |
| *MCW* | 0.26 | 0.13 | 0.17 | *MCW* | -0.12 | -0.24 | 0.05 |
