# Supplementary material for: The Evolution of Morphospace in Phytophagous Scarab Chafers: No Competition - No Divergence?
Source: PLoS One. 2014 May 29;9(5):e98536. doi: 10.1371/journal.pone.0098536 (PMC4038600; doi:10.1371/journal.pone.0098536)
Supplement: Table S5 — PCA-loadings for PCs 1–3 of the analysis of subset 3. BBPM-size-corrected (corr.) and uncorrected dataset (uncorr.). (PDF) [file pone.0098536.s010.pdf]

Table S5. PCA-loadings for PCs 1-3 of the analysis of subset 3. BBPM-size-corrected (corr.) and uncorrected dataset (uncorr.).

**uncorr.**	**PC1**	**PC2**	**PC3**	**corr.**	**PC1**	**PC2**	**PC3**
EL	0.23	0.06	-0.05	*EL*	-0.09	0.05	-0.28
PL	0.2	0.12	-0.09	*PL*	-0.08	0.11	-0.02
Eld	0.24	0.08	-0.09	*Eld*	-0.11	0.09	-0.25
Elmb	0.22	0.01	0.04	*Elmb*	-0.02	-0.03	-0.36
EW	0.22	0.06	-0.28	*EW*	-0.06	0.28	-0.09
Ewb	0.22	0.07	-0.23	*Ewb*	-0.07	0.24	-0.06
PW	0.22	0.08	-0.2	*PW*	-0.08	0.21	-0.08
BH	0.22	0	-0.22	*BH*	-0.01	0.22	-0.13
EH	0.25	0.13	-0.44	*EH*	-0.17	0.43	0.02
HW	0.22	0.05	0.15	*HW*	-0.06	-0.14	0.04
IOD	0.22	0.02	0	*IOD*	-0.03	-0.01	0.28
ED	0.23	0.11	0.19	*ED*	-0.13	-0.19	-0.06
PTL	0.24	0.24	0.39	*PTL*	-0.26	-0.38	0.06
PFL	0.22	0.05	0.26	*PFL*	-0.06	-0.26	0.04
PFW	0.2	0	0.13	*PFW*	0.01	-0.12	0.31
MTL	0.26	0.02	0.34	*MTL*	-0.08	-0.35	-0.04
MTW	0.23	-0.23	-0.25	*MTW*	0.19	0.23	0.6
MFL	0.23	-0.04	0.3	*MFL*	0.01	-0.3	0.02
MFW	0.21	-0.25	-0.03	*MFW*	0.22	0.02	0.27
MCW	0.16	-0.87	0.08	*MCW*	0.87	-0.08	-0.27
