# Supplementary material for: The Evolution of Morphospace in Phytophagous Scarab Chafers: No Competition - No Divergence?
Source: PLoS One. 2014 May 29;9(5):e98536. doi: 10.1371/journal.pone.0098536 (PMC4038600; doi:10.1371/journal.pone.0098536)
Supplement: Table S6 — PCA-loadings for PCs 1–3 of the analysis of subset 4. BBPM-size-corrected (corr.) and uncorrected dataset (uncorr.). (PDF) [file pone.0098536.s011.pdf]

**Table S6. PCA-loadings for PCs 1-3 of the analysis of subset 4**. BBPM-size-corrected (corr.) and uncorrected dataset (uncorr.).

| uncorr. | PC1 | PC2 | PC3 | corr. | PC1 | PC2 | PC3 |
|---|---|---|---|---|---|---|---|
| *EL* | 0.22 | -0.08 | 0.02 | *EL* | -0.09 | 0.03 | 0.28 |
| *PL* | 0.17 | -0.02 | 0.1 | *PL* | -0.08 | 0.21 | 0.13 |
| *Eld* | 0.22 | -0.07 | 0.06 | *Eld* | -0.08 | 0.07 | 0.24 |
| *Elmb* | 0.22 | -0.15 | -0.01 | *Elmb* | -0.15 | -0.01 | 0.25 |
| *EW* | 0.2 | 0.12 | 0.19 | *EW* | 0.07 | 0.26 | 0.14 |
| *Ewb* | 0.2 | 0.1 | 0.2 | *Ewb* | 0.04 | 0.25 | 0.08 |
| *PW* | 0.2 | 0.06 | 0.17 | *PW* | 0.01 | 0.22 | 0.13 |
| *BH* | 0.21 | 0.11 | 0.11 | *BH* | 0.08 | 0.16 | 0.19 |
| *EH* | 0.23 | 0.1 | 0.51 | *EH* | 0.06 | 0.43 | -0.08 |
| *HW* | 0.22 | -0.17 | -0.01 | *HW* | -0.16 | -0.06 | -0.06 |
| *IOD* | 0.23 | -0.08 | 0.18 | *IOD* | -0.07 | 0.07 | -0.46 |
| *ED* | 0.22 | -0.14 | -0.2 | *ED* | -0.11 | -0.2 | 0.23 |
| *PTL* | 0.22 | -0.44 | -0.11 | *PTL* | -0.4 | -0.21 | -0.13 |
| *PFL* | 0.23 | -0.24 | -0.03 | *PFL* | -0.21 | -0.12 | -0.16 |
| *PFW* | 0.22 | -0.1 | 0.11 | *PFW* | -0.1 | 0.06 | -0.28 |
| *MTL* | 0.25 | -0.21 | -0.3 | *MTL* | -0.13 | -0.39 | -0.09 |
| *MTW* | 0.26 | 0.41 | 0.17 | *MTW* | 0.44 | 0.11 | -0.46 |
| *MFL* | 0.24 | -0.14 | -0.25 | *MFL* | -0.07 | -0.31 | -0.1 |
| *MFW* | 0.24 | 0.34 | -0.19 | *MFW* | 0.37 | -0.14 | -0.1 |
| *MCW* | 0.25 | 0.5 | -0.55 | *MCW* | 0.58 | -0.42 | 0.27 |
