# Supplementary material for: The Evolution of Morphospace in Phytophagous Scarab Chafers: No Competition - No Divergence?
Source: PLoS One. 2014 May 29;9(5):e98536. doi: 10.1371/journal.pone.0098536 (PMC4038600; doi:10.1371/journal.pone.0098536)
Supplement: Table S7 — PCA-loadings for PCs 1–3 of the analysis of subset 5. BBPM-size-corrected (corr.) and uncorrected dataset (uncorr.). (PDF) [file pone.0098536.s012.pdf]

Table S7. PCA-loadings for PCs 1-3 of the analysis of subset 5. BBPM-size-corrected (corr.) and uncorrected dataset (uncorr.).

**uncorr.**	**PC1**	**PC2**	**PC3**	**corr.**	**PC1**	**PC2**	**PC3**
EL	-0.21	-0.07	0.11	*EL*	-0.06	-0.18	-0.17
PL	-0.17	0.08	-0.06	*PL*	0.11	-0.15	-0.18
Eld	-0.21	-0.04	0.13	*Eld*	-0.02	-0.19	-0.13
Elmb	-0.22	-0.13	0.16	*Elmb*	-0.12	-0.18	-0.03
EW	-0.19	0.22	0.04	*EW*	0.24	-0.16	-0.17
Ewb	-0.19	0.2	0.07	*Ewb*	0.22	-0.16	-0.09
PW	-0.19	0.15	0.09	*PW*	0.17	-0.17	-0.09
BH	-0.21	0.13	0.08	*BH*	0.13	-0.12	-0.15
EH	-0.24	0.36	0.53	*EH*	0.35	-0.28	0.27
HW	-0.22	-0.15	0.05	*HW*	-0.15	-0.04	0.04
IOD	-0.23	0.05	0.28	*IOD*	0.04	-0.05	0.57
ED	-0.22	-0.23	-0.22	*ED*	-0.23	0.06	-0.51
PTL	-0.22	-0.42	0.06	*PTL*	-0.42	-0.06	0.07
PFL	-0.23	-0.21	0.16	*PFL*	-0.21	-0.06	0.25
PFW	-0.22	0.01	-0.02	*PFW*	0	0.08	0.3
MTL	-0.26	-0.33	-0.08	*MTL*	-0.35	0.18	0.09
MTW	-0.27	0.48	-0.34	*MTW*	0.43	0.51	0.04
MFL	-0.25	-0.23	-0.1	*MFL*	-0.25	0.17	0.08
MFW	-0.23	0.18	-0.51	*MFW*	0.15	0.47	-0.16
MCW	-0.26	0	-0.3	*MCW*	-0.03	0.37	-0.02
