# Supplementary material for: The Evolution of Morphospace in Phytophagous Scarab Chafers: No Competition - No Divergence?
Source: PLoS One. 2014 May 29;9(5):e98536. doi: 10.1371/journal.pone.0098536 (PMC4038600; doi:10.1371/journal.pone.0098536)
Supplement: Table S8 — Percentage of total variation explained by principal components summing up to ≥95%. BBPM-size-corrected and uncorrected dataset. (PDF) [file pone.0098536.s013.pdf]

Table S8. Percentage of total variation explained by principal components summing up to ≥95%. BBPM-size-corrected and uncorrected dataset.

	uncorrected					size-corrected										
**PC axis**	**1**	**2**	**3**	**4**	**5**	**1**	**2**	**3**	**4**	**5**	**6**	**7**	**8**	**9**	**10**	**11**
**Complete**	86.9	4.6	2.0	1.6		34.9	15.5	11.9	8.9	7.6	4.2	4.0	3.0	2.1	1.9	1.4
**Subset 1**	88.5	4.1	3.0			36.0	23.5	14.4	8.6	4.4	3.5	2.7	1.9	1.4		
**Subset 2**	85.5	4.9	3.7	2.0		47.1	20.5	10.8	6.5	4.5	3.6	2.0				
**Subset 3**	89.5	3.9	1.5			38.5	13.7	11.3	7.6	6.2	4.4	4.1	3.8	2.0	1.8	1.7
**Subset 4**	88.5	2.8	2.2	1.4	1.1	23.4	19.1	12.4	9.3	8.5	6.1	5.3	3.9	2.6	2.2	1.9
**Subset 5**	88.5	3.1	1.9	1.5	1.1	24.9	16.8	12.7	9.9	8.2	7.3	5.6	3.5	2.5	2.2	1.7
