# Supplementary material for: The Evolution of Morphospace in Phytophagous Scarab Chafers: No Competition - No Divergence?
Source: PLoS One. 2014 May 29;9(5):e98536. doi: 10.1371/journal.pone.0098536 (PMC4038600; doi:10.1371/journal.pone.0098536)
Supplement: Table S9 — The impact of size. Percentage of variation explained by size alone (PVESA) within the subsets. (PDF) [file pone.0098536.s014.pdf]

Table S9. The impact of size. Percentage of variation explained by size alone (PVESA) within the subsets.

**Taxa subset**	**PVESA (%)**
1	88.4
1*	82.4
2	85.2
3	89.5
4	88.5
5	88.4

* without *Microvalgus*
