# Supplementary material for: The Evolution of Morphospace in Phytophagous Scarab Chafers: No Competition - No Divergence?
Source: PLoS One. 2014 May 29;9(5):e98536. doi: 10.1371/journal.pone.0098536 (PMC4038600; doi:10.1371/journal.pone.0098536)
Supplement: Table S10 — Alternative size correction: F-values from non-parametric MANOVA of the complete sampling (excluding singletons) regarding 95% of total variation. Values for the size-corrected dataset (with linear regression) are shown in the upper triangle, those for the uncorrected in the lower one. Significant differences (p<0.05) are highlighted in bold. Higher F-values for the same significant pairings are underlined in the respective triangle. (PDF) [file pone.0098536.s015.pdf]

Table S10. Alternative size correction with linear regression: F-values from non-parametric MANOVA of the complete sampling (excluding singletons) regarding 95% of total variation. Values for the size-corrected dataset are shown in the upper triangle, those for the uncorrected in the lower one. Significant differences ($p<0.05$) are highlighted in bold. Higher F-values for the same significant pairings are underlined in the respective triangle.

	Adoretini	Anomalini	Aphodiinae	Cetoniini	Clade B	Dynastinae	Glaphyridae	Hopliinae	Hybosoridae	Scarabaeinae	Sericini A	Sericini B	Sericini C	SWM	Valgini
Adoretini		5.34*	15.35*	15.18*	**8.93**	12.65*	5.05*	3.95*	4.06*	5.42*	**13.49**	**19.60**	**19.12**	6.34*	6.37*
Anomalini	5.22*		19.02*	**7.86**	**6.17**	**4.60**	4.53*	5.50*	3.00*	6.97*	**6.28**	**8.22**	**6.94**	2.30	3.86*
Aphodiinae	39.30*	47.75*		11.78*	**10.50**	15.75*	5.72	15.36*	3.94	7.72	19.39*	**35.60**	**30.30**	7.89*	8.08
Cetoniini	7.73*	1.29	33.83*		**11.15**	9.18*	3.49*	10.67*	5.33*	8.25*	9.44*	**15.75**	**10.90**	3.16*	3.06
Clade B	3.96	0.88	**21.07**	0.55		**8.35**	3.00*	**6.63**	1.55	10.18*	**14.36**	**35.66**	**21.80**	2.42*	5.76*
Dynastinae	13.83*	3.12	49.12*	0.45	0.98		8.47*	12.72*	2.57	7.47*	**6.92**	**14.28**	**11.91**	3.84*	3.98*
Glaphyridae	0.39	1.41	24.58	2.10	1.01	4.37		2.83*	5.43	3.84	7.39*	**8.84**	7.58*	1.60	5.75
Hopliinae	1.30	8.40*	17.05*	9.73*	6.21*	15.44*	1.18		4.43*	5.78*	**13.97**	**18.53**	**16.35**	4.31*	5.81*
Hybosoridae	2.87	6.29*	13.29	5.92	3.02	9.76*	5.89	0.56		2.82	5.88*	**8.34**	7.70*	1.45	4.31
Scarabaeinae	5.13	0.81	30.33	0.47	0.38	1.13	1.93	5.16	8.10		10.67*	**16.29**	14.56*	5.32*	2.42
Sericini A	8.01*	20.27*	17.92*	**22.65**	**19.12**	**30.91**	4.81*	3.77*	1.63	11.26*		3.81*	3.93*	4.98*	5.71*
Sericini B	2.58	9.99*	**24.01**	**16.18**	**32.11**	**21.02**	1.56	2.64	1.51	5.19*	3.90*		1.76	**9.74**	7.79*
Sericini C	5.51*	**18.89**	**17.44**	**23.88**	**30.56**	**30.92**	3.17	2.51	1.24	8.68*	0.47	4.64*		**5.48**	6.78*
SWM	0.43	3.28	5.76*	5.05*	7.61*	6.50*	0.35	0.24	0.14	1.47	1.10	1.19	1.22		2.45*
Valgini	4.83	9.63*	0.82	8.25	8.61*	10.72*	1.59	2.09	0.69	2.69	2.82	7.14*	3.56	1.57	

* Significant without sequential Bonferroni correction.
