# Supplementary material for: The Evolution of Morphospace in Phytophagous Scarab Chafers: No Competition - No Divergence?
Source: PLoS One. 2014 May 29;9(5):e98536. doi: 10.1371/journal.pone.0098536 (PMC4038600; doi:10.1371/journal.pone.0098536)
Supplement: Table S11 — Alternative size correction: F-values from non-parametric MANOVA (Anderson 2001) of each subset (ss1–ss5, excluding singletons) regarding 95% of total variation. Values for the size-corrected dataset (with linear regression) are shown in the upper triangle, those for the uncorrected in the lower one. Significant differences (p<0.05) are highlighted in bold. (PDF) [file pone.0098536.s016.pdf]

Table S11. Alternative size correction with linear regression: F-values from non-parametric MANOVA (Anderson 2001) of each subset (ss1-ss5, excluding singletons) regarding 95% of total variation. Values for the size-corrected dataset are shown in the upper triangle, those for the uncorrected in the lower one. Significant differences (p<0.05) are highlighted in bold.

Subset 1	Cetoniinae	Clade A	
Cetoniinae		**8.66**	
Clade A	0.87		
Subset 2	Adoretini	Anomalini	Dynastinae
Adoretini		**5.60**	**12.97**
Anomalini	**0.02**		**4.96**
Dynastinae	**0.00**	0.10	
Subset 3	Clade B	Clade C	
Clade B		**29.17**	
Clade C	**41.85**		
Subset 4	Sericini	SWM	
Sericini		**9.27**	
SWM	1.08		
Subset 5	Sericini A	Sericini B	Sericini C
Sericini A		**3.73**	**3.97**
Sericini B	3.82*		2.03
Sericini C	0.56	4.58*	

* Significant without sequential Bonferroni correction.
