# Supplementary material for: The Evolution of Morphospace in Phytophagous Scarab Chafers: No Competition - No Divergence?
Source: PLoS One. 2014 May 29;9(5):e98536. doi: 10.1371/journal.pone.0098536 (PMC4038600; doi:10.1371/journal.pone.0098536)
Supplement: Table S12 — Alternative size correction: Correlation between molecular and morphometric distance-matrices for specimens within one feeding type and the complete sampling (size-correction with linear regression). Coefficients of determination and p-values from Mantel-tests. (PDF) [file pone.0098536.s017.pdf]

**Table S12. Alternative size correction with linear regression: Correlation between molecular and morphometric distance-matrices for specimens within one feeding type and the complete sampling.** Coefficients of determination and p-values from Mantel-tests.

| | r | p |
|---|---|---|
| complete | 0.06 | < 0.01 |
| ANT[1] | 0.25 | < 0.01 |
| COP[2] | 0.48 | 0.21 |
| HERB[3] | 0.43 | < 0.01 |
| SFU[4] | 0.29 | 0.21 |
| SAP[5] | -0.30 | 0.82 |

[1]anthophilous, [2]coprophagous, [3]herbivorous, [4]sap / fluid utilizers, [5]saprophagous
