# Supplementary material for: The Evolution of Morphospace in Phytophagous Scarab Chafers: No Competition - No Divergence?
Source: PLoS One. 2014 May 29;9(5):e98536. doi: 10.1371/journal.pone.0098536 (PMC4038600; doi:10.1371/journal.pone.0098536)
Supplement: Table S13 — Results of the phylogenetic least squares analyses. Coefficients of determination and p-values are given for the uncorrected and both size-corrected data sets. (PDF) [file pone.0098536.s018.pdf]

Table S13. Results of the phylogenetic least squares analyses. Coefficients of determination and p-values are given for the uncorrected and both size-corrected data sets.

data set	r^2	p
uncorrected	0.03	0.65
size-corrected		
BBPM	0.03	0.06
linear regression residuals	0.06	< 0.01
